# Evaluating the Use of In-Game Rule Changes as a Primary Prevention Approach to Reduce Injury Risk in Invasion Team Sports: A Scoping Review

**DOI:** 10.1007/s40279-026-02405-8

**Published:** 2026-03-12

**Authors:** Hamish Gornall, Haley Truscott, Isla J. Shill, Mike Ashford, Debbie Palmer

**Affiliations:** 1 Edinburgh Sports Medicine Research Centre, Institute for Sport, PE and Health Sciences, University of Edinburgh, Edinburgh, UK; 2 UK Collaborating Centre on Injury and Illness Prevention in Sport, Edinburgh, UK; 3 Sports Injury Prevention Research Centre, Faculty of Kinesiology, University of Calgary, Calgary, Canada

## Abstract

**Background:**

The rate of injury in invasion team sports is often greater than in non-invasion sports. A preventative measure to reduce injury risk is to implement in-game rule changes aimed at modifying game events or player behaviour.

**Objective:**

This study reviewed the use of in-game rule changes in invasion team sports with the aim of reporting the level of utilisation, effectiveness, and unintended consequences.

**Design:**

Scoping review.

**Data Sources:**

Scopus, MEDLINE, SPORTDiscus, CINAHL, Web of Science.

**Results:**

In total, 2205 studies were identified, 116 full texts were screened and 47 were included in the final review. Seven sports were found to have assessed in-game rule changes with the most common being ice hockey (*n* = 18), tackle football (*n* = 11) and rugby union (*n* = 7). Rule changes were found to have had the intended effect in 28 studies, while nine found no change and four reported an increase in the rate or cause of injury. The unintended consequences associated with a rule change were assessed by four studies with a further two evaluating several rule changes across multiple seasons.

**Conclusions:**

In-game rule changes were investigated in half of the sports included in the search. Evidence suggests rule changes can be an effective method for reducing injury risk, although unintended consequences need to be considered. Rule changes should be evaluated in a variety of contexts, with an emphasis being placed on researching female populations to address the extensive knowledge gap.

**Supplementary Information:**

The online version contains supplementary material available at 10.1007/s40279-026-02405-8.

## Key Points

**Table:** 

The effects of in-game rule changes were only found to have been investigated in seven of the 14 sports searched for.
In-game rule changes identified commonly included removing inciting events from gameplay, altering the rules of an in-game event, or increasing the level of sanctions for foul play or head contact.
A greater emphasis must be placed on researching the effects of in-game rule changes on female, woman and girl athletes as only one study was found to have evaluated this population exclusively.

## Introduction

Invasion team sports require participants to engage in regular player-on-player collisions such as tackling, body checking (BC) and blocking. These movements are executed in high-speed dynamic environments where players must be physically and mentally proficient to perform these skills effectively whilst also minimising their risk of injury [1]. Unfortunately, owing to the frequency and intensity associated with these collisions, injuries still occur and often at a greater rate compared with non-invasion sports [2, 3]. For example, the rate of severe injuries is far greater in men’s ice hockey [IR (injury rate) = 2.84/1000 athletic exposures (AE)] versus men’s baseball [IR = 0.65/1000 AE] [4]. Likewise, there is a higher rate of injury in women's football (soccer) [IR = 2.23/1000 AE] compared to tennis [IR = 0.97/1000 AE] [4].

Minimising the risks associated with player-on-player collisions through preventative interventions is an important factor to maintaining the positive health outcomes associated with team sports [5, 6]. The van Mechelen sequence of prevention model is often utilised when attempting to reduce injury risk as it informs prevention practice through a cyclical model of surveillance, intervention design, implementation and evaluation [7]. Sports including ice hockey, tackle football (American and Canadian) and football have adopted this model when introducing an in-game rule change and reported positive outcomes as a result of targeting specific in-game events that have a propensity to cause injury [8, 9]. A widely reported example of this in the literature is the removal of BC in Canadian youth ice hockey leagues [10]. A BC is a manoeuvre where the defending player uses their upper body to force the attacking player off the puck [11], and disallowing this in-game contact event for players under the age of 12 resulted in a 51% and 60% reduction in the overall injury and sport related concussion (SRC) rates, respectively [10].

In-game rule changes appear to be underutilised in sport injury prevention, potentially due to a resistance towards altering core aspects of gameplay or dismissive attitudes regarding the importance of player safety and injury prevention [12]. Either way, scrutiny of the injury and SRC rates across invasion sports is increasing and finding a balance between acceptable levels of risk and prevention is key. In-game rule changes may offer an effective solution to address this concern yet there is limited cross-sport evaluation assessing the utilisation, effectiveness and unintended consequences of these attempts to reduce sport related injuries [13, 14]. This scoping review aims to address this by i) summarising the use of in-game rule change injury prevention measures across invasion team sports, ii) asses the effectiveness of in-game rule changes to reduce sport related injury risk and iii) identify the unintended consequences and limitations of this approach to inform future research.

## Methods

A scoping review was deemed appropriate as this study aimed to 1) establish the extent of research in this area and 2) identify the gaps in knowledge that currently exist [15]. The review followed Arksey and O’Malley’s [16] five stage process and incorporated the recommendations set out in the Levac et al. [17] framework. The study is reported in accordance with the PRISMA-Scoping Review guidelines (Supplementary Material 1) [18].

### Stage 1—Identifying the Research Questions

An initial literature search helped inform the research questions. The primary aim of the review was to provide greater context surrounding the use of in-game rule changes in invasion team sports as a means of preventing sport related injuries. The research questions were:

- To what extent are in-game rule changes being utilised in invasion sports to reduce injury risks?
- Are in-game rule changes an effective approach to reduce invasion team sports injury risk?
- What are the unintended consequences and limitations of in-game rule changes for injury prevention?

### Stage 2—Identifying Relevant Studies

An initial search on Scopus identified key words and terms applicable to answering the research questions. The invasion sport definition was informed by work from Hopper and Butler et al. [19] where the essential components and intentions of sporting games (e.g., target, striking, net/wall or invasion) were mapped out and defined. The current review included sports that require participants to kick, throw or hit an object towards a designated area or goal while an opposing team is actively attempting to defend the area and regain possession. The electronic search was conducted using five databases including Scopus, MEDLINE, CINAHL, SPORTDiscus and Web of Science [20]. A citation search was carried out during the full text review. The first database search was conducted in January 2024 and re-run in December 2024 and November 2025.

### Stage 3—Study Selection

The inclusion and exclusion criteria were developed by assessing previous publications (Table 1) [14, 21, 22]. Owing to the extensive variation in methodological approaches, consideration was only given if the intervention evaluated the effectiveness or unintended consequences of an in-game rule change that reduced, altered or impacted injury rates or risk factors (e.g., head-to-head contacts or illegal collisions). Secondary reviews (e.g., scoping or systematic reviews) were excluded as they may disregard outcome measures required to comprehensively answer the present study's research questions. Grey literature was not included as it lacks a formal peer review process and may contain discrepancies or unvalidated methodological approaches [23].

**Table 1:** Inclusion and exclusion criteria

Inclusion criteria	Exclusion criteria
Studies evaluating invasion games	Assessment of injury interventions for batting, fielding, net, wall, target and leisure sports or activities
Studies measuring the effectiveness or unintended consequences of an in-game rule change that reduced, altered or impacted a participants injury risk	Evaluation of prevention methods including training and education programs, or protective equipment mandates
Studies reporting competitive match play and training injuries	Laboratory research, modelling studies, meta-analyses, scoping or systematic reviews, grey literature, opinion pieces or magazines
Studies using a control and intervention or comparison group design to establish the effectiveness pre- and post-rule change	Studies assessing intervention adoption rates or cost-effectiveness
Studies reporting quantitative outcomes including number/rate, point and effect estimates of injuries or risk factors (e.g., head-to-head contacts or illegal actions)	Studies not originally written in English

Reviewers H.G. and H.T. met to discuss the inclusion and exclusion criteria prior to title and abstract screening. Each reviewer independently assessed titles and abstracts; the reviewer agreement was 95.6% (H.G./H.T.) [24]. Disagreements were resolved through further discussion. Upon completion, an independent full text review for final inclusion was undertaken alongside a citation search to identify any missed relevant studies. Authors were contacted when full texts could not be found. Reviewer agreement for full text screening was 82.3% (H.G./H.T.). Disagreements were resolved through further discussions. A third reviewer was not required for either screening stage.

### Stage 4—Data Charting

Reviewers H.G. and H.T. designed the charting form and tested it prior to data extraction to ensure the research questions could be answered. H.G. and H.T. jointly extracted two randomly selected articles to develop an understanding of the process. Once completed, both reviewers independently extracted data from the first three studies to determine if extraction was consistent. H.G. extracted data from 90% of the included studies, while H.T. extracted data from the remaining 10%. H.G. checked 10% of H.T. studies and vice versa and minimal discrepancies were identified so no additional cross checking was undertaken.

### Stage 5—Collating, Summarising, and Reporting Results

The extracted data are reported on the basis of the sport and sub-categorised by the type of in-game rule change (e.g., disallowing bodychecking, increased sanctions, etc.). The descriptive data reported for each study included:

- Player sex
- Level of competition
- Age group
- Sample size
- Study design
- Data collection method
- Rule change description
- Injury or risk factor point estimates
- Injury or risk factor effect estimates
- Supplementary details regarding the study's aims, outcomes, unintended consequences and limitations were extracted to provide wider context. This information is available in Supplementary Material 3.

## Results

### Search Analysis

**Fig. 1:**
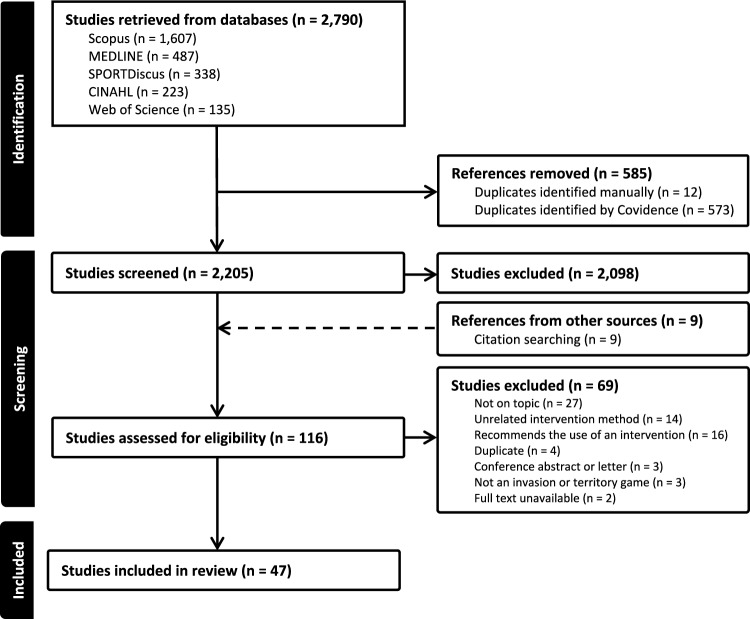
Preferred reporting items for systematic reviews and meta-analyses. Scoping review flowchart

The initial database search (January 2024) returned 2188 studies; updated searches were conducted in December 2024 and November 2025 with a further 602 articles added. After 585 duplicates were removed, 2205 studies remained. The title and abstract review ruled out 2089 studies, leaving 116 studies for full text review (107 retained from title and abstract review and nine additional studies identified from citation searches). Full text review deemed 69 studies to be ineligible. Overall, 47 studies met the inclusion criteria (Fig. 1).

### Study Characteristics

The search yielded 47 studies across seven invasion sports consisting of 18 (38%) ice hockey studies, 11 (24%) tackle football, seven (15%) rugby union, seven (15%) football, two (4%) Australian Rules football, one (2%) rugby league and one (2%) lacrosse (Table 2). Research was predominantly conducted on male athletes with this population being investigated in 35 (75%) studies. A further 11 (23%) studies reported on a mixed population, only three (6%) of which presented disaggregated male and female injury rates. There was only one (2%) study included in the review that focused exclusively on female athletes. A total of 23 (49%) studies investigated youth and adolescent athletes while the remaining 24 (51%) reported on adult populations.

**Table 2:** Study characteristics

Characteristics (n = 47 studies)	*N* (%)
*Publication Year*	
1990–1999	3 (6)
2000–2009	3 (6)
2010–2019	15 (32)
2020–2024	26 (56)
*Sport*	
Ice hockey	18 (38)
Tackle football	11 (24)
Rugby union	7 (15)
Football	7 (15)
Australian Rules football	2 (4)
Rugby league	1 (2)
Lacrosse	1 (2)
*Study Type*	
Cohort-Retrospective	31 (66)
Cohort-Prospective	15 (32)
Cross sectional	1 (2)
Sex	
Male-only	35 (75)
Female-only	1 (2)
Male and female	11 (23)
*Study demographic*	
Adolescent (10–18 years)	21 (45)
Adult (> 18)	24 (51)
Adolescent and youth (8–18 years)	2 (4)
*Level*	
Amateur	29 (62)
Elite	17 (36)
Mixed	1 (2)
*Data collection method*	
Injury surveillance forms	15 (32)
Hospital/Medical/Insurance records	10 (21)
Open access injury data	7 (15)
Video analysis	6 (13)
Sport governing body data	1 (2)
Multiple collection methods	8 (17)

Of the 47 included studies, 17 (36%) reported rule changes significantly reduced the rate or cause of injury or SRC [10, 11, 25–39], four (9%) reported a reduction in the rate of injury or SRC but did not report corresponding effect estimates [40–43], whilst a further seven (15%) studies reported a reduction in the rate of in-game events linked to injury or SRC (e.g., head contacts and foul play) [44–50]. Of the 28 studies reporting an effective in-game rule change, three of these studies also identified unintended consequences including increased injury severity [10, 35], and transferring the injury risk to an alternative match event [34]. There were six (13%) studies reporting no significant change to either the rate or cause of injury after introducing a rule change [51–56] and three (6%) that failed to report a reduction in the rate of injuries or SRC after seeing the intended player behaviour change [57–59]. Four (8%) studies reported a significant increase in the rate of injury [60–63] and a further four (8%) that directly explored the unintended consequences of an in-game rule change [64–67]. Finally, two (4%) studies reported on the outcome of several injury prevention rule changes over multiple seasons [68, 69]. Full details of all included studies are provided in Supplementary Material 3.

### Ice Hockey

Of the 18 studies evaluating ice hockey, 12 investigated male athletes while 6 examined a mixed population. There were 14 rule change studies at amateur level and 4 in an elite setting with studies predominantly focusing on the permitted age of BC and reducing head-to-head contact. Five studies evaluating adolescent BC rules found rates of injury, SRC and head contacts significantly increased for players in leagues where BC was permitted, compared with leagues where BC was disallowed [10, 35, 48, 64, 67]. Emery et al. [11, 25] evaluated this rule in 13–14 and 15–17-year-old cohorts. In leagues where BC was disallowed, they reported a reduction in injury and SRC rates for both the 13–14 [incidence rate ratio (IRR) = 0.45 (95% CI 0.27–0.77); SRC IRR = 0.59 (95% CI 0.31–1.17)] and 15–17 [IRR = 0.30 (95% CI 0.19–0.46); SRC IRR = 0.43 (95% CI 0.24–0.76)]-year-old players [11, 25]. Conversely, one of the studies investigating the unintended consequences of reducing the legal age of BC from players aged 12–13 down to age 9–10 years found no increase in the rate of injury [65]. Watson et al. [40] investigated a rule penalising players who bodychecked their opponent from behind in collegiate hockey leagues: effect estimates were not reported although the rate of head and neck injuries decreased [40].

Five studies evaluated rules prohibiting players from making direct contact to an opponent’s head. Hutchinson et al. [26] evaluated the rule in an elite adult cohort and found no significant change in the rate of SRC but did find a reduction in the rate of SRC caused by blindside bodychecking (i.e. a player is unaware of the BC prior to being struck) [26]. A similar rule change evaluated in a youth cohort initially failed to reduce the rate of head contacts [pre amendment IRR = 0.94 (0.76–1.15)] [56]. After an additional policy amendment where the level of sanction was further increased, the rate of head contacts reduced [IRR = 0.70 (95% CI 0.51–0.95)] [44]. The remaining two studies reported a significant increase in head contact or SRC as a result of implementing the head contact rule change [60, 63]. Three studies evaluated rules increasing the level of sanctions for actions including fighting, repetitive fouling and dangerous BC with all three reporting reductions in either injury or concussion incidence rate [27, 28, 41]. Morrissey et al. [68] evaluated multiple rule changes designed to reduce the rate of SRC. While rates were observed to increase, the authors suggested this may have been a result of improved SRC education, recognition and reporting [68].

### Tackle Football

All 11 tackle football studies were conducted on male athletes, with 8 studies investigating adult populations. Eight studies [29, 30, 37, 43, 47, 51, 61, 66] evaluated rule changes designed to lower the rate of head collisions. These included altering special teams plays (kick-offs (KO) and punts), banning targeting (deliberately lowering the crown of the helmet to initiate contact) and implementing a mercy rule (activating a rolling game clock in the second half when a score differential has been met to reduce athletes’ exposure time). Of these eight studies, six found a reduction in either the rate or cause of SRC [29, 30, 37, 43, 47, 66]. Baker et al. [66] and Westerman et al. [61] specifically investigated the unintended consequences of the lowering of the helmet/targeting rule. They hypothesised the rule change may cause an increase in lower extremity (LE) injuries. At elite level in the National Football League (NFL), Baker et al. [66] reported a significant reduction in the rate of SRC [IRR = 0.60 (0.50–0.73)] while LE injuries [IRR = 0.97 (95% CI 0.92–1.04)] and severe LE injuries [IRR = 1.15 (95% CI 0.99–1.33)] did not significantly increase [head contact 66]. Westermann et al. [61] found the implementation of the targeting rule in the collegiate game did not have the intended effect as the rate of SRC increased [IRR = 1.34 (95% CI 1.08–1.66)]. The rule also unintentionally increased LE time loss injuries [IRR = 1.21 (95% CI 1.06–1.36)] [61]. One study assessed a KO rule change that aimed to reduce the rate of SRC by increasing the fair catch zone from the 20 to 25-yard line [51]. The rule change did not significantly alter the rate of SRC [IRR = 1.89 (95% CI − 1.22 to 5.01)] [51].

The three remaining studies assessed knee, spinal, SRC, and overall injury rates [31, 36, 42]. Baker et al. [31] evaluated a rule disallowing the chop block, an action where the offensive player dives off their feet towards a defending player's leg in an attempt to bring them to the ground. The rate of knee injuries significantly reduced [IRR = 0.84 (95% CI 0.75–0.96)] after introducing this rule [31]. Torg et al. [42] evaluated a rule change designed to remove spear tackling and headfirst blocking. No effect estimates were reported although the rate of spinal trauma injuries for high school and collegiate athletes reduced by 70% and 65%, respectively [42]. Ruestow et al. [36] evaluated a KO rule change aimed at reducing the overall rate of injuries and SRC. The rule change significantly reduced the rate of KO injuries for the KO team [IRR = 0.45 (95% CI 0.28–0.73) but not the receiving team [IRR = 0.75 (95% CI 0.41–1.08)]. No significant difference was found regarding the rate of SRC for the KO team [IRR = 0.49 (95% CI 0.13–1.81)] [36].

### Rugby Union

The search identified seven rugby union studies. Males were investigated in five studies while females and a mixed population were researched in the remaining two [38, 49, 52, 53, 57–59]. A scrum engagement law change aiming to reduce spinal injuries was investigated in two studies [38, 52]. The first, in 2007, implemented a touch and pause prior to scrum engagement. This ensured there was a standardised distance between the two forward packs, thus aiming to lower the impact forces through the players' spines upon scrum engagement. The rate of spinal injuries did decrease, although not significantly [IRR = 0.79 (95% CI 0.53–1.18)] [52]. The law was adapted again in 2010, further reducing the distance, speed and force between the opposing packs. The study evaluated the law change in French amateur rugby and reported a 91% reduction in scrum related spinal injuries [38].

To reduce the rate of head-to-head contacts and SRC, five studies evaluated lowering the maximum legal height of the tackle [49, 53, 57–59]. There were three studies assessing a reduction from the shoulders to below the armpit [53, 58, 59]. In an elite male setting this law had the desired effect on player behaviour as 15% fewer tackles were executed above the armpit. This change did not cause a reduction in the rate of SRC overall [IRR = 1.31 (95% CI 0.85–2.01)], and it also unintentionally increased tackler SRC rates [IRR = 1.90 (95% CI 1.05–3.45)] [59]. The same law variation was trialled in both a university [53] and schoolboy [58] setting, with no significant reductions in the rate of SRC for either the university players [IRR = 0.69 (95% CI 0.40–1.10)] or schoolboys [U18 IRR = 0.95 (95% CI 0.59–1.47)] [53, 58]. The remaining two studies assessed a lowered tackle height law variation from the shoulder down to the base of the sternum [49, 57]. In the men’s game, there was a significant decrease in head-to-head contacts for the tackler [IRR = 0.55 (95% CI 0.33–0.92)] but not for the ball carrier [IRR = 0.61 (95% CI 0.36–1.05)] [49]. In the women's game head-to-head proximity decreased for the tackler [IRR = 0.71 (95% CI 0.62–0.82)] and ball-carrier [IRR = 0.67 (95% CI 0.58–0.77)], but the overall rate of SRC remained unchanged (IRR = 1.11 (95% CI 0.50–2.43) [57].

### Football (Soccer)

There were seven football studies, five in male and two in mixed populations. The rules being evaluated aimed to reduce the rate or cause of head contacts, SRC, collisions and overuse injuries [34, 39, 45, 46, 50, 54, 62]. Three studies in the elite men’s game assessed increasing the level of sanction for an aerial collision (AC) that resulted in elbow to head contact [34, 45, 50]. Beaudouin et al. [34, 50] reported a reduction in both head injuries [IRR = 0.71 (95% CI 0.57–0.86)] and elbow to head contacts (23% less), but this did not correlate to a significant reduction in SRC [IRR = 0.71 (95% CI 0.46–1.09)] [34]. Bjørneboe et al. [45] assessed the same rule change in a different elite men’s league and found a significant reduction in the rate of head contacts [IRR = 0.81 (95% CI 0.67–0.99)]. Shibukawa et al. [46] assessed a rule allowing goalkeepers to restart the game with a short pass in their own penalty box, with the intention that it may lead to less longball kicks and ACs. This change reduced the rate of high-risk free ball ACs by 23% and goal kick ACs by 36% [46].

Kriz et al. [54] assessed a yellow card policy enforcing player suspensions for accumulating multiple cards over a set time period. The rule change had no impact on contact injuries [Male: IRR = 1.10 (95% CI 0.99–1.22); Female: IRR = 1.02 (95% CI 0.92–1.13)] or combined male and female SRC rates [IRR = 1.03 (95% CI 0.88–1.22)] [54]. Lalji et al. [62] evaluated a rule disallowing 10–13-year-old male and female players from heading the football in an attempt to reduce SRC. The study reported a significant rise in the odds of SRC [odds ratio (OR) = 1.29 (95% CI 1.09–1.52)] [62]. However, the authors failed to control for confounding factors such as increased SRC awareness and reporting which could be contributing to the increased SRC rate. Qureshi et al. [39] investigated the impact of a rule allowing elite teams to use a maximum five substitutions rather than three. This rule likely helped reduce the odds of a player having multiple injuries [OR = 0.68 (95% CI 0.56–0.82)] or a muscle injury [OR = 0.72 (95% CI 0.59–0.87)] over the course of the season.

### Australian Rules Football

Two studies evaluating in-game rule changes in men’s elite Australian rules football were included [33, 69]. Aimed at reducing the rate of posterior cruciate ligament (PCL) injuries, a rule limiting the run-up distance of ruck men at centre bounce (equivalent to a KO) was introduced and evaluated [33]. The rule significantly reduced the rate of PCL knee injuries [Relative risk (RR) = 0.16 (95% CI 0.04–0.69)]. A second Australian rules study reviewed eight rule changes designed to lower the overall rate of injuries. The rule changes were effective in targeting and reducing PCL knee injuries [RR = 0.16 (95% CI 0.04–0.69)], head and neck injuries [RR = 0.72 (95% CI 0.57–0.91)] and leg injuries [Groin: RR = 0.76 (95% CI 0.62–0.93); Hamstring: RR = 0.81 (95% CI 0.70–0.93)] [69]. The five remaining rule changes introduced did not alter the rate of SRC or lower limb injuries.

### Rugby League

A single study was identified in rugby league [32]. A law change limiting teams to 12 interchangeable substitutions rather than an unrestricted quantity was evaluated in the sub-elite men’s game. The rule change caused a reduction in the overall rate of injuries [IRR = 0.70 (95% CI 0.65–0.75)] [32].

### Lacrosse

Only one lacrosse study was identified, evaluating two in-game rule changes [55]. The rule changes included disallowing BC to defenceless players and penalising intentional head contact. The rule changes did not alter the overall rate of injuries [IRR = 0.95 (95% CI 0.85–1.06)], BC-related injuries [IRR = 0.92 (95% CI 0.75–1.13)] or SRC injuries [IRR = 0.98 (95% CI 0.60–1.60)] in competitive games [55].

### Discussion

Evaluations of in-game rule changes that reduced, altered or impacted the rate or cause of injury were identified across seven invasion team sports. The rule changes often removed an in-game event from gameplay, altered the rules of an in-game event or increased the level of sanctions for foul play or head contacts. Rule changes reported having the intended effect in 28 of the included studies, although three of these studies did also identify unintended consequences associated with altering a rule. There were seven studies that found no significant difference in the rate or cause of injury, while four studies reported a significant increase after introducing an in-game rule change. The increased injury risk was often attributed to poor implementation, a lack of adherence or an ineffective design. Concerningly, the review only identified one study that investigated a female population, demonstrating the lack of research and knowledge gap that exists between male and female athletes.

### Design and Implementation

One of the most effective rule changes has been the removal of BC in Canadian youth ice hockey. This rule change has led to a significant reduction in overall injury, head contacts and reported SRC [11, 25, 48], although it still remains controversial amongst players and parents [70]. This controversy stems from the notion that removing BC for 9–12-year-old players may just delay injury risk, and cause BC injuries later in the participation cycle owing to a lack of skill proficiency [64, 67]. Studies investigating this theory found little evidence to support the skill proficiency argument as the risk of BC injuries in older players did not decrease as a result of BC experience. These findings have been further ratified by Eliason et al. [71] where the author group determined a reduction in the rate of SRC caused by a player having BC experience did not offset the number of concussions that have been prevented by banning the manoeuvre outright.

Discussions similar to those in youth ice hockey have emerged in rugby union, with calls to ban tackling in youth and adolescent age groups [72]. This suggestion has gained traction as the tackle event is responsible for the majority of injuries and SRC [73]. Owing to the frequent and physical nature of the tackle event, contact proficiency is recognised as a key factor for reducing players injury and SRC risk [74, 75]. Therefore, removing the opportunity for youth players to develop this skill may not only increase future injury risk but also impact participation rates. A law change such as this would drastically alter a fundamental aspect of game play and risk alienating players, coaches and fans [76, 77]. A more realistic and widely accepted approach is to adapt the current laws or rules around the execution of the event, such as the previously trialled and now implemented lowered tackle height law variation in amateur rugby union [49, 53, 57].

Lowering the tackle height from the shoulder to the armpit and then the base of the sternum in rugby union has had mixed results regarding the risk of concussion. Research in both elite mens and schoolboy populations found players behaviour aligned with the intention of the law variation, with bent at waist tackling increasing by 15% and 34% respectively. Yet, these behaviour changes did not cause a meaningful reduction in SRC in schoolboy rugby and unintentionally increased SRC rates in the elite men’s game [58, 59]. The tackle height was further lowered to the base of the sternum for a minimum of two seasons in amateur rugby [78]. Studies exploring the first season of the height reduction reported less head-to-head and head-to-shoulder contact and proximity, although SRC rates remained unchanged [57]. Encouragingly, longitudinal injury surveillance from an Irish cohort has linked sternum and below tackling to a 33% and 18% reduction of SRC in men’s and woman’s rugby over the 2-year trial period [79]. Lowering the tackle height to the base of sternum for multiple seasons has hinted towards a more effective outcome. This demonstrates the importance of giving players time to adapt their behaviour ensuring they become proficient at executing the action in the desired manner [49, 80].

### Unintended Consequences

Adaptation of contact events such as tackling, bodychecking or blocking requires extensive work to ensure any unintended consequences can be minimised. For example, the lowering of the helmet/targeting rule introduced in elite and collegiate tackle football reported differing results. In the NFL, there was a reduction in the rate of SRC while LE injury rates remained largely unchanged [43, 66]. Conversely, the collegiate population saw an increase in both SRC and LE injuries with the author hypothesising players dropped their tackle height to avoid being penalised and inadvertently increased the rate of LE injuries [61]. While the parameters of the rule change were similar for both collegiate and NFL players, the reason behind the differing outcomes are likely multifactorial. Factors including tackle proficiency, playing experience, level of play and rule enforcement all play a role. An additional consideration effecting the reported SRC rates are the socio-cultural differences between collegiate and NFL teams, where the likelihood of a player self-reporting a concussion may differ [81].

Rule changes designed to reduce the rate of concussion or head injuries were evaluated in six of the included sports, yet several studies found no change, and in some circumstances SRC rates increased. A common hypothesis for the unexpected increase is the ongoing improvement in SRC awareness, identification and reporting in contact sports [26, 60–62, 68]. While socio-cultural barriers to reporting concussions still exist [82], there does appear to have been a shift in knowledge and attitude, which is a positive development for contact sports. This has likely stemmed from ongoing research and awareness campaigns including SRC education programs in both elite and amateur sport [83–85]. These improvements do present a challenge when attempting to differentiate between the true impact of a rule change versus extraneous factors (e.g., increased SRC education and improved reporting tendencies). A means of addressing this is through the implementation of routine injury surveillance and video analysis. Collectively, these data provide a baseline understanding, allowing for meaningful comparisons to be made between control and intervention cohorts as well as helping to identify any unintended consequences [7].

### Evaluation

Several approaches were used to evaluate the outcome of an in-game rule change with injury surveillance being the most common. Capturing injuries and exposure in this manner can be challenging, especially in an amateur setting where there tends to be a lack of resources [86]. The data collection responsibilities can regularly fall on coaches, team managers and physiotherapists, who often operate in a voluntary capacity and may not believe in the need or have the time to devote to reporting injuries [87]. Furthermore, medical cover within female amateur sports can be scarce or non-existent [88]. This was evident in the present review where a single study investigated rule changes in female only sporting population. An alternative approach used by a quarter of the studies was to assess hospital or insurance records [27, 30, 38, 52, 62, 64, 65, 67, 68]. This method is not as resource intensive for researchers and can provide greater accuracy regarding injury diagnosis [89]. A limitation of this approach is these records are entirely dependent on the severity of an athlete’s injury and whether it necessitates professional medical attention [90]. Therefore, this approach tends to limit studies in reporting only severe injuries, causing mild or moderate injuries to go unreported [91]. Importantly, hospital records may also lack sport specific injury context and athletes’ exposure time, meaning it is impossible to determine the injury mechanism, severity or burden [91].

The optimum rule change evaluation approach is to pair injury surveillance data with corresponding video footage [92]. This approach was used by five studies in the review, and it often led to a better overall understanding when assessing the impact of the rule change [26, 45, 57–59]. Adopting this method does require extensive resources including camera equipment, injury report forms and video analysis software, and thus is not always a viable option in community sport [93]. If unfeasible, future rule change evaluations should aim to have a routine injury surveillance system in place prior to and during the evaluation stage. Furthermore, male and female athletes should be investigated separately as injury outcomes can vary due to the intrinsic differences (e.g., height, weight, aerobic capacity and muscle strength) between these populations [94–96]. Implementing these approaches will help to ensure in-game rule changes are assessed in a comprehensive manner across a variety of sporting contexts.

### Rule Change Limitations

An ineffective in-game rule change was often due to an inadequate implementation [59, 69], a lack of adherence [54, 56, 63] or a suboptimal design [53, 58, 68, 69]. To reduce the likelihood of a poor outcome, an intervention first requires evidenced-based decision-making, often informed by either the van Mechelen sequence of prevention or Translating Research into Injury Prevention Practice (TRIPP) models [7, 97]. Following this, gaining 'buy-in' from stakeholders (e.g., coaches, players, parents and match officials) is an important step, as these individuals are partially responsible for actioning the change [13, 20]. Achieving this requires early consultation to understand the varied contexts and attitudes towards injury risk and prevention [98, 99]. Furthermore, when disseminating and evaluating a new rule change, maintaining a strong dialogue between the organisation orchestrating the intervention and the stakeholders administering it is pivotal for continued adherence [54, 59].

Evaluating the design and identifying any unintended consequences is also a crucial step. Organisations must be prepared to continually assess rule changes, and in cases where surveillance data indicates an ineffective outcome, amendments should be made to ensure athletes' safety is not compromised [38, 44, 49, 53, 57]. Finally, using an in-game rule change alone may not always be the optimum approach when addressing an injury concern. A multifaceted strategy where neuromuscular training programs, and protective equipment mandates are used in conjunction with rule adaptations may provide a more effective outcome [20].

### Study Limitations

Study inclusion criteria were kept broad in regard to publication dates, injury definitions, methodological approach and reported data. This ensured all relevant literature was included and the key research questions could be addressed. There are however drawbacks with this approach, the main one being a lack of quality assessment. This resulted in the inclusion of studies that contained small sample sizes or missing point/effect estimates. Results must therefore be interpreted with caution as low statistical power and overestimated or missing effect sizes may have potentially skewed the perceived effectiveness of some rule changes [14, 100]. Another limitation was the high degree of variation in methodological approach between studies and across each sport. This discrepancy was commonly caused by the evolution in research practices, often precipitated by the publication of injury surveillance reporting consensus statements. This added a layer of complexity when assessing the data as it was not always possible to categorise study outcomes and the opportunity to directly compare rule changes to one another was limited [101].

## Conclusions

This scoping review identified 47 studies spanning seven sports that assessed in-game rule changes, such as the elimination or alteration of specific events to mitigate participants' injury risk. Rule changes were assessed in youth and adult populations and predominantly focused on male athletes as only one study researched females. Rule changes were deemed effective in 28 studies, with the removal of high-risk in-game events often having the greatest effect. However, this approach may not always be appropriate as it can alter core principles of game play. To improve future research, studies should align with the sequence of prevention or TRIPP model [7, 97] and ensure robust injury surveillance is in place across both male and female populations. This will allow for a comprehensive evaluation and ensure changes in athletes' behaviour can be monitored in a longitudinal manner.

## Supplementary Information

Below is the link to the electronic supplementary material.

Supplementary file1 (DOCX 38 KB)

Supplementary file2 (DOCX 111 KB)

## Declarations

### Data Availability Statement

All data relevant to the study are reported in the text or included in the supplementary material.
