# Supplementary material for: Evaluating the Use of In-Game Rule Changes as a Primary Prevention Approach to Reduce Injury Risk in Invasion Team Sports: A Scoping Review
Source: Sports Med. 2026 Mar 12;56(4):927–39. doi: 10.1007/s40279-026-02405-8 (PMC13124859; doi:10.1007/s40279-026-02405-8)
Supplement: Supplementary file 2 — Supplementary file2 (DOCX 111 KB) [file 40279_2026_2405_MOESM2_ESM.docx]

| **Titles and Acronyms** | **Description** |
| --- | --- |
| Injury Surveillance forms  Hospital/medical/insurance records  Open access injury data Video Analysis  NGB/School Database Combined Approach | Injury data recorded by an individual associated with a team and returned for the purpose of injury surveillance. Data obtained from hospital, medical or insurance databases for injuries caused by sporting participation. Data obtained through open access sources such as news reports, team injury reports, and online public sources. Data obtained by analysing video footage of game play injuries.  Data obtained from national injury databases using data collected by governing bodies, schools or governments. A combination of methods utilised to assess injuries (e.g. Injury surveillance forms and video analysis). |
| Clinical Treatment (CT)  Medical Attention (MA)  Time Loss (TL) | Received treatment for an injury from a qualified clinician. Received medical attention at any point after the injury occurred.  Missed time participating as a result of the injury (24-h, training session, 7-day, game loss). |
| SRC  IR  IRR/RR  OR | Sport Related Concussion Incidence Rate  Incident rate ratio/Relative Risk (*Where possible rate ratios not reported in text were calculated post data extraction).  Odds Ratio |
| BC KO  KR  DQ | Bodychecking  Kick Off Kick Return  Disqualification  Highschool |
| HS |  |

**Evaluating the use of in-game rule changes as a primary prevention approach to reduce injury risk in invasion team sports: A scoping review.**

**Supplementary Material 3 – Study Summary Table**

**Sports Medicine Open
Authors:** Hamish Gornall^1,2^, Haley Truscott^4^, Isla J. Shill^1,2,4^, Mike Ashford^3^, Debbie Palmer^1,2,4^

**Author Affiliations:**

1. Edinburgh Sports Medicine Research Network, Institute for Sport, PE and Health Sciences, Moray House School of Education and Sport, University of Edinburgh, Edinburgh, United Kingdom
2. UK Collaborating Centre on Injury and Illness Prevention in Sport, United Kingdom
3. Moray House School of Education and Sport, University of Edinburgh, Edinburgh, United Kingdom
4. Sports Injury Prevention Research Centre, Faculty of Kinesiology, University of Calgary, Canada

Corresponding Author email address – h.gornall@sms.ed.ac.uk

| **Study Details**  Sport (Location)  Reference | **Population**  Sex  Level  Age  Measure + Sample size | **Study Design**  Data collection method | **Rule Change**  In-game event targeted.  Injury site targeted.  Rule definition/study aim. | **Primary Outcome Measures**  Injury definition used.  Reporting measure (injuries or cause). | **Control Rates**  **95% CI** (unless otherwise stated) | **Intervention Rates**  **95% CI** (unless otherwise stated) | **Effect Estimates**  **95% CI** (unless otherwise stated) | **Study Outcomes**  Rule change outcome  Unintended consequences  (As reported by authors) | **Study Limitations**  (As reported by authors) |
| --- | --- | --- | --- | --- | --- | --- | --- | --- | --- |
| **Ice Hockey**  (Canada)  Hagel et al. (2006) | Male and female  Amateur  Adolescent  Injured players, pre (n = 90), post (n = 159)  Injuries extrapolated using player population and registration denominators. | **Retrospective Cohort**  Hospital/medical/insurance records. | **Rule – BC**  **Injuries – Overall**  Determining the effects of a divisional rule reducing the legal age of BC from 12 years to 11 years old. | **Injury Definition** MA   **Measure**  Incidence Rate.  Severe Injuries (SRC, head injury, fractures, neck sprains, hospital admission). | **Injury** N = 90  40.6/1,000 11-year-old players  **Severe Injuries**  n = 33 (40.2%)  **SRC Injuries**  n = 6 (6.7%) | **Injury** N = 159  85.5/1,000 11-year-old players  **Severe Injuries**  n = 77 (51%)  **SRC Injuries**  n = 20 (12.6%) | **Injury RR** IRR = 1.9 (1.4 to 2.4) Male IRR = 2.1 (1.6 to 2.8)  Female IRR = 0.6 (0.2 to 1.5)  **Severe Injury RR**  IRR = 2.4 (1.6 to 3.6)  **SRC Injury RR** IRR = 3.4 (1.4 to 8.4) | **Outcomes**  IR was 2x greater for children aged 11 when allowing them to BC.  No future injury reduction benefit attributed to early adoption of BC as injuries and SRC increased. | **Limitations**  Hospital reports were inconsistent.  League specific rules were unavailable.  11-year-old players may have been subject to increased injury risk, going from the oldest players in the division (10-11 years) to being the youngest (11-12 years). |
| **Ice Hockey**  (Canada)  Cusimano et al. (2011) | Male  Amateur  Children + Adolescent (6-17 yrs.)  Atom division injuries, pre (n = 518), post (n = 495) | **Retrospective Cohort**  Hospital/medical/insurance records. | **Rule – BC**  **Injuries - Overall**  Determining the effects of reducing the legal age of BC from age 11 to age 9 in the atom division. | **Injury Definition**  Clinical Treatment  **Measure**  BC Injuries.  BC SRC Injuries.  BC Head/Neck. Injuries. | **BC Injuries**  Atom = 158  **BC SRC Injuries**  Atom = 4  **BC Head/Neck Injuries**  Atom = 55 | **BC Injuries**  Atom = 243  **BC SRC Injuries**  Atom = 22  **BC Head/Neck Injuries**  Atom = 95 | **BC Injuries**  Atom OR = 2.20 (1.70 to 2.84), p = <0.05  **BC SRC Injuries**  Atom OR = 10.08 (2.35 to 43.29), p = 0.01  **BC Head/Neck Injuries**  Atom OR = 2.27 (1.42 to 3.65), p = 0.001 | **Outcomes**  Introducing BC at age 9 instead of age 11 significantly increased the rate of injuries and SRC.  No future injury reduction benefit attributed to early adoption of BC as injuries and SRC increased across all older adolescent divisions.  Rule change proved to be dangerous and therefore reversed due to effect on the IR. | **Limitations**  Data collection limited to hospital reports.  Specific age of injured players was unavailable therefore OR were calculated using division age bands (e.g. Atom). |
| **Ice Hockey**  (Canada)  Black et al. (2016) | Male and female  Amateur  Adolescent  Players - Control (n = 590), Intervention (n = 281) | **Prospective Cohort**  Injury surveillance forms. | **Rule – BC**  **Injuries - Overall**  Comparison of youth leagues (11-12 yrs.) allowing and disallowing BC. | **Injury Definition** 24-h TL  **Measure**  Incidence Rate.  SRC rate.  Severe SRC (>10-day TL). | **Incidence Rate (No BC league)**  1.6/1,000 player hours (0.87 to 2.94)  **SRC (No BC league)**  0.91/1,000 player hours (0.41 to 2.06)  **Severe SRC**  0.91/1,000 player hours (0.58 to 1.42)  **SRC Avg Days Lost**  No BC = 12.5 days | **Incidence Rate (BC league)**  4.19/1,000 player hours (3.38 to 5.19)  **SRC (BC league)**  2.78/1,000 player hours (2.19 to 3.52)  **Severe SRC**  0.46/1,000 player hours (0.15 to 1.42)  **SRC Avg Days Lost** BC = 5 days | **BC Injury RR** IRR = 2.62 (1.38 to 4.96)  **SRC Injury RR** IRR = 3.04 (1.31 to 7.03)  **Severe SRC RR**  IRR = 0.45 (-0.13 to 1.04) | **Outcomes**  There was a 3-fold increase of injury and SRC when competing in a league permitting BC.  **Unintended consequences** SRC severity was higher in leagues disallowing BC, although occurred 3x less often. | **Limitations**  Sample size limited due to available resources for collecting data.  Injuries <7 days reported by team delegate rather than trainer or physician.  Potential overestimate regarding SRC lasting <7 days due to MA not being sought. |
| **Ice Hockey**  (Canada)  Black et al. (2017) | Male and female  Amateur  Adolescent  Players - Control (n = 883), Intervention (n = 618) | **Prospective Cohort**  Injury surveillance forms. | **Rule - BC**  **Injury – Overall**  Prohibit BC for players aged 11-12. | **Injury Definition**  24-h TL  MA  **Measure**  Incidence rate.  SRC rate. | **Incidence Rate** 4.37/1,000 game hours (3.59 to 5.33)  **SRC** 2.79/1,000 game hours (2.28 to 3.41)  **SRC MA**  Control = 67% | **Incidence Rate** 2.16/1,000 game hours (1.56 to 2.99)  **SRC**  1.12/1,000 game hours (0.75 to 1.69)  **SRC MA**  Intervention = 80%. | **Incidence RR**  IRR = 0.49 (0.34 to 0.72)  **SRC Injury RR**  IRR = 0.40 (0.26 to 0.63)    **BC Injury RR**  IRR = 0.16 (0.08 to 0.32)  SRC IRR = 0.21 (0.09 to 0.45) | **Outcomes**  Significant reduction in injury, SRC, and BC injuries for intervention group.  **Unintended consequences**  Potential increase in SRC severity. | **Limitations**  Small sample size due to limited data collection ability of the study. |
| **Ice Hockey**  (Canada)  Kolstad et al. (2022) | Male  Amateur  Adolescent  Games – Control (n = 13), Intervention (n = 13) | **Retrospective Cohort**  Video analysis | **Rule – BC**  **Injuries – Head and trunk**  Comparison of BC league vs non-BC league on rate of head and trunk contacts. | **Injury Definition**  Head-to-head and trunk contact  **Measure**  HC rates  BC Trunk contact (level 4 = heavy and intense contact, level 5 = deliberate and excessive contact) | **U15 Level 4 BC**  4.44/100 player minutes (3.69 to 5.35)  **U15 Level 5 BC**  0.48/100 player minutes (0.30 to 0.77)  **U18 Level 4 BC**  2.19/100 player minutes (1.58 to 3.02)  **U18 Level 5 BC**  0.42/100 player minutes (0.28 to 0.64)  HC rate not reported | **U15 Level 4 BC**  0.82/100 player minutes (0.62 to 1.11)  **U15 Level 5 BC**  0.05/100 player minutes (0.01 to 0.23)  **U18 Level 4 BC**  0.40/100 player minutes (0.24 to 0.66)  **U18 Level 4 BC**  0.01/100 player minutes (0.01 to 0.13)  HC rate not reported | **U15 Level 4 BC**  IRR = 0.19 (0.13 to 0.26)  **U15 Level 5 BC**  IRR = 0.11 (0.03 to 0.51)  **U18 Level 4 BC**  IRR = 0.18 (0.10 to 0.33)  **U18 Level 5 BC**  IRR = 0.04 (0.01 to 0.31)  **U15 HC**  IRR = 0.40 (0.22 to 0.71)  **U18 HC**  IRR = 0.37 (0.25 to 0.57) | **Outcomes**  Overall trunk contact was lower in the non-BC U15 and U18 leagues.  Overall BC was significantly less in the non-BC U15 and U18 leagues.  Overall HC was significantly less in the non-BC U15 and U18 leagues.  The contact intensities (level 2 to 5) were less in the non-BC leagues. | **Limitations**  Video analysis could not capture all contact events and therefore some events were missed and not part of the analysis.  Difference out with BC could be present between the compared divisions 3-6 but were not explored. |
| **Ice Hockey**  (Canada)  Emery et al. (2020) | Male and female  Amateur  Adolescent  Players – BC league (n = 608), non-BC league (n = 396) | **Prospective Cohort**  Injury surveillance forms. | **Rule – BC**  **Injuries - Overall** Prohibit BC in nonelite 13–14-year-old leagues. | **Injury Definition**  MA + TL  **Measure** Incidence rate.  Severe Injuries (>7 days). Severe SRC (>10 days). | **Injuries** 5.52/1000 player hours (3.03 to 8.01)  >7-day 3.34/1000 player hours (1.41 to 5.27)  **SRC Injuries**  2.31/1000 player hours (0.49 to 4.13)  **Severe SRC Injuries**  1.33/1000 player hours (0.00 to 2.83) | **Injuries**  2.50/1000 player hours (0.20 to 4.80)  >7-day 1.29/1000 player hours (0.00 to 2.94)  **SRC Injuries**  1.37/1000 player hours (0.00 to 3.30)  **Severe SRC Injuries**  0.73/1000 player hours (0.00 to 2.25) | **Injury RR** IRR = 0.45 (0.27 to 0.77)  >7-day 0.39 (0.22 to 0.71)  **SRC Injury RR**  IRR = 0.59 (0.31 to 1.17)  **Severe SRC Injuries**  0.55 (0.24 to 1.41) | **Outcomes**  The rule changes reduced the IR, SRC rate, and 7 and 10-day TL injuries.  Greatest difference in IR was among younger populations (11-12 years). | **Limitations**  Not all SRC injuries received clinical follow ups.  Playing position not accounted for.  Data collection limited due to individual team resources. |
| **Ice Hockey**  (Canada)  Emery et al. (2022) | Male and female  Amateur  Adolescent  Players – Control (n= 674), Intervention (n = 453) | **Prospective Cohort**  Injury surveillance forms. | **Rule – BC**  **Injuries - Overall** Prohibit BC for 15-17-year-old players. | **Injury Definition**  MA + TL  **Measure** Incidence rate.  Severe Injuries (>7 days). Severe SRC (>10 days). | **Injuries** 8.17/1000 player hours (6.33 to 10.01)  >7-day 4.69/1000 player hours (3.46 to 6.59) **SRC Injuries**  2.65/1000 player hours (1.84 to 3.45)  **Severe SRC Injuries**  1.57/1000 player hours (0.91 to 2.24) | **Injuries** 2.65/1000 player hours (1.27 to 4.03)  >7-day 0.33/1000 player hours (0.00 to 0.86) **SRC Injuries**  1.19/1000 player hours (0.49 to 2.24)  **Severe SRC Injuries**  0.07/1000 player hours (0.00 to 0.25) | **Injury RR** IRR = 0.30 (0.19 to 0.46)  >7-day 0.07 (0.03 to 0.17)  **SRC Injury RR**  IRR = 0.43 (0.24 to 0.76)  **Severe SRC Injuries**  0.04 (0.01 to 0.30) | **Outcomes**  67% and 59% reduction in injury and SRC rate respectively, attributed to the removal of BC.  The effect of the BC rule change was greater in the 15–17-year-old division compared to 13–14-year-old division.  Greater reduction in injury within 15–17-year-old division possibly caused by differences in player size, speed and aggression. | **Limitations**  Not all SRC injuries were clinical checked.  Return to play procedures may have been affected by game importance (e.g. playoffs).  Socioeconomic factors not accounted for. |
| **Ice Hockey**  (Canada)  Kukaswadia et al. (2010) | Male  Amateur  Children + Adolescent (7-14 years)  Injuries (n =  2,554) | **Retrospective Cohort**  Hospital/medical/insurance records. | **Rule - BC**  **Injuries – Overall**  Reduced the legal age of BC from 12-13yrs down to 9-10yrs. | **Injury Definition**  Clinical Treatment  **Measure**  Incidence rate.  BC Incidence rate.  SRC & Head injury. | **Incidence Rate (Pre age reduction)**  59.9/1,000 player years (55.4 to 64.4)  **BC Incidence Rate (Pre age reduction)**  18.7/1,000 player years (16.1 to 21.3)  **Head Injury** n = 79/640 (12.3%)  **BC Head Injury**  n = 30/200 (15%) | **Incidence Rate (Post age reduction)**  49.1/1,000 player years (44.8 to 53.3)  **BC Incidence Rate (Post age reduction)**  17.2/1,000 player years (14.7 to 19.8  **Head Injury**  n = 62/484 (12.8%)  **BC Head Injury**  n = 25/170 (14.7%) | **Head Injury**  p = 0.82  **BC Head Injury**  p = 0.94 | **Outcomes**  No increase in injury.  Additional rule changes for illegal head contact likely to influence the outcome.  Improved coaching may have contributed to lowering the IR. | **Limitations**  Height and weight of players unaccounted for.  Self-reporting injuries may be inaccurate.  Declining use of emergency departments may have contributed to a perceived reduction in the IR. |
| **Ice Hockey**  (Canada)  Watson et al. (1996) | Male  Amateur  Adult  Injuries (n = 653)  Penalties (n = 389) | **Prospective cohort**  Injury surveillance forms | **Rule – BC**  **Injuries – Head and upper body**  Penalising checking players from behind | **Injury Definition**  MA  **Measure**  Incidence rates. | **Head/Neck**  6.16/1,000 player games  **Back**  4.98/1,000 player games  **Shoulder**  16.11/1,000 player games | **Head/Neck**  4.49/1,000 player games  **Back**  4.49/1,000 player games  **Shoulder**  19.38/1,000 player games | **Effect estimates not reported in text** | **Outcomes**  Reduction in head/neck injuries after the rule change.  Overall, a safer hockey environment was created. | **Limitations**  Study did not report any limitations |
| **Ice Hockey**  (United States)  Hutchison et al. (2023) | Male  Elite  Adult  Games (n = 4,920) | **Retrospective Cohort**  Injury surveillance forms.  Video analysis. | **Rule - BC**  **Injuries – Head**  Prohibit direct contact to the head when blindside bodychecking. | **Injury Definition**  Clinical Treatment  **Reported Measure**  SRC rate.  BC SRC. Blindside SRC (Unaware of BC prior to being hit). | **Blindside BC SRC**  1.6/100 games  **SRC Rate** 6.1/100 games  **BC SRC Rate**  2.3/100 games | **Blindside BC SRC**  1.0/100 games  **SRC Rate**  8.3/100 games  **BC SRC Rate**  2.4/100 games | **Blindside BC SRC**  0.6/100 games (90% CI 0.30 to 0.90)  **SRC**  2.2/100 games (90% CI 1.0 to 3.0)  **BC SRC**  0.1/100 games (90% CI -0.4 to 0.5) | **Outcomes**  The rule change did have the intended effect of reducing the rate of blindside SRC.  The overall rate of SRC did increase after the rule change.  SRC increase partially attributed to improved education regarding recognise and remove procedures. | **Limitations**  Not all events were coded due to poor video footage.  Only reviewed regular season injury events (post season excluded). |
| **Ice Hockey**  (Canada)  Williamson et al. (2021) | Male  Elite  Adolescent  Games – pre policy (n = 16), post policy (n = 16) | **Retrospective Cohort**  Video analysis | **Rule – Gameplay**  **Injuries - Head** Zero tolerance for HC policy – unintentional HC = 2 min penalty, intentional HC = 4- or 5-min penalty plus game misconduct or ejection. | **Injury Definition**  Head-to-head contact  **Measure** Direct HC1 - player inflicted.  Indirect HC2 - environment (head hits boards, glass, or ice). | **Direct HC1 (Pre)**  N = 166  **Indirect HC2 (Pre)**  N = 95  **HC (Pre)**  16.6/100 team min | **Direct HC1 (Post)**  N = 175  **Indirect HC2 (Post)**  N = 70  **HC (Post)**  15.5/100 team min | **Direct HC1**  IRR = 1.05 (0.86 to 1.28)  **Indirect HC2**  IRR = 0.74 (0.50 to 1.11)  **Overall HC**  IRR = 0.94 (0.76 to 1.15) | **Outcomes**  No significant difference in HC1, HC2 or overall HC between the two cohorts.  BC mechanism was predominantly associated with HC.  More enforcement by match officials is required to benefit from the rule change. | **Limitations**  The study could only evaluate HCs and not concussions.  Not all physical contacts and HC were captured on video.  Video quality improved between the pre and post rule change years potentially effecting the coding quality. |
| **Ice Hockey**  (Canada)  Williamson et al. (2022) | Male  Elite  Adolescent  Games – Pre policy (n = 10), post policy (n = 8), further policy amendments (n = 10). | **Prospective Cohort**  Video analysis. | **Rule – Gameplay**  **Injuries - Head** Zero tolerance for HC policy – sanction increased to a 5 min penalty plus misconduct call for players incurring either 3 HC penalties, causing injury or cross checking a player above the shoulders. | **Injury Definition**  Head-to-head contact  **Measure** Direct HC1 - player inflicted.  Indirect HC2 - environment (head hits boards, glass, or ice). | **Direct HC1 (Pre)** 11.66/100 team min (10.26 to 13.25)    **Indirect HC2 (Pre)**  6.16/100 team min (4.73 to 8.02) | **Direct HC1 (Post)** 12.73/100 team min (12.36 to 15.65)    **Indirect HC2 (Post)**  5.09/100 team min (3.16 to 8.22)  **Direct HC1 (Amendment)**  8.88/100 team min (7.02 to 11.24)  **Indirect HC2 (Amendment)**  7.45/100 team min (5.98 to 9.27) | **Direct HC1 RR (pre to amendment)**  IRR = 0.76 (0.58 to 0.99)  **Indirect HC2 RR (pre to amendment)** IRR = 1.21 (0.86 to 1.70)  **Direct HC1 RR (post to amendment)**  IRR = 0.70 (0.51 to 0.95)  **Indirect HC2 RR (post to amendment)**  IRR = 1.46 (0.87 to 2.45) | **Outcomes**  The initial rue change showed little effect, although policy amendments resulted in direct HC being reduced by 30%.  Indirect head contact did not differ as a result of the rule change.  Direct HC penalty counts did not differ due to the rule change.  Enforcement of rules was inconsistent and required further work. | **Limitations**  Video does not capture all incidents as they may have taken place out of frame.  Difference in camera quality effected the accuracy of the analysis. |
| **Ice Hockey**  (Canada)  Donaldson et al. (2013) | Male  Elite  Adult  Players (n =2,211) | **Retrospective Cohort**  Open access injury data. | **Rule – Gameplay**  **Injuries - Head** Rule 48 Prohibiting direct contact to an opposition players head in season 2010/11 and 11/12. | **Injury Definition** MA  **Measure** SRC Rate (Rates measured from law change year 2010/11). | **SRC Injuries**  2009/10 = 3.58/100 games | **SRC Injuries** 2010/11 = 5.28/100 games  2011/12 = 6.83/100 games | **SRC Injury RR** 2009/10 compared to 2010/11 IRR = 0.64 (0.42 to 0.96)  2011/12 compared to 2010/11 IRR = 1.35 (0.96 to 1.89) | **Outcomes**  SRC injuries increased after the introduction of the law change in the elite league.  Additional seasons are required to determine the impact.  Enforcement of the rule requires addressing to ensure player behaviour change. | **Limitations**  Access to professional medical records unavailable.  The study did not account for previous SRC history. |
| **Ice Hockey**  (Canada)  Krolikowski et al. (2017) | Male and female  Amateur  Adolescent  Players - Control (n = 1,269), Intervention (n = 830) | **Retrospective Cohort**  Injury surveillance forms. | **Rule – BC**  **Injury – Head**  Prohibit direct contact to the head when BC in Pee Wee (11-12 yrs.) and Bantam (13-14 yrs.) divisions. | **Injury Definition**  Clinical Treatment  **Measure**  SRC.  Severe SRC (>10-day TL). | **SRC 11-12yrs**  n = 58 (2007-08)  **Severe SRC**  n = 13 (2007-08)  **SRC 13-14yrs**  n = 23 (2008-09)  **Severe SRC**  n = 5 (2008-09) | **SRC 11-12yrs**  n = 74 (2011-12)  **Severe SRC**  n = 35 (2011-12)  **SRC 13-14yrs**  n = 37 (2011-12)  **Severe SRC**  n = 24 (2011-12) | **SRC 11-12yrs RR**  IRR = 1.85 (1.20 to 2.86)  **Severe SRC RR**  IRR = 4.12 (2.00 to 8.50)  **SRC 13-14yrs RR**  IRR = 2.48 (1.17 to 5.24)  **Severe SRC RR**  IRR = 7.91 (3.13 to 19.94) | **Outcomes**  Increased rate of SRC after the rule change.  Increased player size and speed may have impacted SRC rates.  SRC increase partially attributed to improved education regarding recognise and remove procedures. | **Limitations**  Injury mechanisms unassessed due to the lack of video analysis.  Generalisability of this rule change to differing levels and ages may be limited. |
| **Ice Hockey**  (United States)  Roberts et al. (1996) | Male  Amateur  Adolescent  Players (n = 273) | **Prospective Cohort**  Injury surveillance forms. | **Rule – Sanctions**  **Injuries - Overall**  Fair play rule – Additional points for not fighting or deliberately trying to injure an opponent. | **Injury Definition**  MA  **Measure** Incidence Rate. Notable IR (SRC, dental, facial, and injuries required professional attention and player withdrawal). | **Injuries (Regular Play)** 294.1/1,000 player hours  **Notable Injuries** 126.1/1,000 player hours  **Penalties**  n = 13 per game | **Injuries (Fair Play)** 89.7/1,000 player hours   **Notable Injuries**  29.9/1,000 player hours  **Penalties**  n = 7.1 per game | **Effect estimates not reported in text** | **Outcomes**  Fair play rules produced fewer injuries, reduced the rate of notable injuries and lowered the quantity of penalties per game. | **Limitations**  Due to the screening definitions for injuries, SRC may have gone unreported.  The difference in players weight and height was considerable, yet unaccounted for in the study. This may have impacted the IR.  Officials’ interpretation of the rules may have impacted the results. |
| **Ice Hockey**  (United States)  Kriz et al. (2019) | Male  Amateur  Adolescent  Games (n = 1,762) | **Retrospective Cohort**  Hospital/medical/insurance records. | **Rule - Sanctions**  **Injuries – Overall**  Suspensions for repeated in game penalty offenses. | **Injury Definition**  Clinical Treatment  **Measure**  Incidence rate.  SRC Rate. | **BC Injuries**  n = 51  **Head/SRC BC Injuries**  n = 27  **DQ Penalties** n = 48 (5.2%) in 923 games | **BC Injuries**  n = 31  **Head/SRC BC Injuries**  n = 13  **DQ Penalties** n = 37 (4.4%) in 839 games | **BC Injuries**  OR = 0.55 (0.35 to 0.86), p = 0.008  **Head/SRC BC Injuries**  OR = 0.44 (0.23 to 0.85), p = 0.012  **DQ Penalties**  OR = 0.84 (0.54 to 1.31), p = 0.440 | **Outcomes**  Significant reduction in upper body and SRC injuries.  A reduction in DQ penalties was seen after the rule change, although this was not significant. | **Limitations**  Hospital injury forms were not consistent, resulting in a likely underreporting of BC injuries.  Additional rule changes addressing BC were introduced during this time period. |
| **Ice Hockey**  (United States)  Nadkarni et al. (2021) | Male  Amateur  Adolescent  Injuries (n = 517) | **Retrospective Cohort**  NGB injury Database. | **Rule – Sanctions**  **Injuries - Overall**  Increased sanctions for dangerous BC and slamming opponents into the rink boards. | **Injury Definition**  Clinical Treatment  **Measure**  Incidence rate | **Incidence Rate** 22.3/10,000 AE (19.8 to 24.9)  **Injuries Delivering BC**  1.0/10,000 AE (0.5 to 1.6)  **Injuries Receiving BC** 8.3/10,000 AE (6.8 to 10.0) | **Incidence Rate** 19.4/10,000 AE (15.9 to 22.2)  **Injuries Delivering BC**  1.5/10,000 AE (0.8 to 2.4)  **Injuries Receiving BC** 5.0/10,000 AE (3.8 to 6.5) | **Incidence RR**  IRR = 0.87 (0.73 to 1.04), p = 0.12  **Injuries Delivering BB**  IRR = 1.52 (0.72 to 3.22), p = 0.27  **Injuries Receiving BC**  IRR = 0.60 (0.43 to 0.83), p = 0.002 | **Outcomes**  Overall IR decreased.  Significant reduction in injuries caused from players receiving a BC.  No significant change to mechanism of injury in control group. | **Limitations**  Only used data provided by athletic trainers.  Could not assess penalties due to a lack of game metric data. |
| **Ice Hockey**  (United States)  Morrissey et al. (2022) | Male  Amateur  Adolescent  SRC (n = 848, sample used to estimate a national rate). | **Retrospective Cohort**  Hospital/medical/insurance records. | **Rule – Multiple**  **Injuries - Head**  Assessment of all rule changes between 2007 to 2011 and 2012 to 2016 regarding SRC. | **Injury Definition**  Clinical Treatment  **Measure**  SRC Rate. | **SRC (2007)**  N = 656  21.84/10,000 person years  **SRC (2012)** N = 1,965 65.30/10,000 person years | **SRC (2011)**  N = 2,043  66.85/10,000 person years  **SRC (2016)** N = 1,292 40.76/10,000 person years | **SRC 2007-11** p = 0.014  **SRC 2012-16** p = 0.036 | **Outcomes**  Overall increase in SRC in youth ice hockey.  Reduction from 2012-16 indicates effective use of rule changes.  SRC increase predominantly attributed to improved education regarding recognise and remove. | **Limitations**  Not all players registered with NGB injury database.  Level of competition was not accounted for. |
| **American Football** (United States)  Wiebe et al. (2018) | Male  Amateur  Adult  KO Plays - pre (n = 2,379), post (n = 1,467) | **Retrospective Cohort**  Injury surveillance forms. | **Rule – KO**  **Injuries – Head** Move KO mark from 30yd to 40yd line. Touchback line from 25-yd line to 20-yd line. | **Injury Definition**  Clinical Treatment  **Measure** KO SRC Rate. | **SRC Injuries** SRC n = 26  SRC = 10.93/1,000 KO plays | **SRC Injuries**  SRC n = 3  SRC = 2.04/1,000 KO plays | **SRC Injuries** SRC Difference n = -23  SRC Difference = -7.51(-12.88 to -2.14)/1,000 KO plays | **Outcomes**  The KO rule change saw significantly less SRC caused by the KO after the rule change.  The average number of touchbacks increased from 17.9% per year to 48.0% per year due to the rule change. | **Limitations**  The impact of additional rule changes was not accounted for within the study. |
| **American Football** (United States)  Baker et al. (2021) | Male  Elite  Adult  Players (n = 1,696) | **Retrospective Cohort**  Open access injury data. | **Rule – Gameplay**  **Injuries – Head**  Article 8 - prohibiting the use of the helmet to contact the opponent. | **Injury Definition** Game Loss  **Measure** SRC Rate.  Average Games missed. | **SRC Injuries** n = 299 SRC / Season  5.5/1000 AE  **Games Missed** n = 1.17 | **SRC Injuries**  n = 180 SRC / Season  3.3/1000 AE  **Games Missed** n = 1.26 | **SRC Injury RR** RR = 0.60 (0.50 to 0.73)  **Games Missed**  P = 0.63 | **Outcomes**  The rule change significantly reduced the rate of SRC.  The rule change did not significantly reduce the number of games missed due to SRC.  Equipment improvements may also be reducing SRC rate.  **Unintended consequence**  Although not assessed, lower extremity injuries require investigation after the introduction of this rule change. | **Limitations**  SRC lasting 2-5 days were not accounted for due to team reporting requirements.  Player education not accounted for when quantifying the rule change.  Validity of collecting open access injury data limited the study. |
| **American Football** (United States)  Obana et al. (2021) | Male and female  Amateur  Adolescent  SRC (n = 4983) | **Retrospective Cohort**  Hospital/medical/insurance records. | **Rule – Gameplay**  **Injuries – Head**  Targeting rule -penalising players for direct head contact. | **Injury Definition** Clinical Treatment  **Measure**  Compare the change in SRC diagnosis rates over 4 years pre to 4 years post rule change. | **SRC Injuries**  SRC diagnosis increased by 10.7% from 2009 to 2013.  Helmet to helmet SRC diagnosis increased by 17.5% from 2009 to 2013. | **SRC Injuries 2015-19**  SRC diagnosis decreased by 6.2% from 2015 to 2019.  Helmet to helmet SRC diagnosis decreased by 5.6% from 2015 to 2019. | **SRC**  SRC diagnosis, p = 0.04.  Helmet to helmet SRC diagnosis, p = 0.03. | **Outcomes**  Significant reduction in overall SRC and head-to-head SRC rates from 4 years pre to 4 years post rule change.  Higher rate of SRC was seen in competitive game play.  SRC was potentially underreported due to the stigma surrounding missing game time. | **Limitations**  Data lacked player position information.  Not all SRC are treated by clinicians therefore data likely underreporting the true rate of SRC.  Results cannot be extrapolated across all levels and competitions. |
| **American Football** (United States)  Baker et al. (2022) | Male  Elite  Adult  Injuries (n = 3,685) | **Retrospective Cohort**  Open access injury data. | **Rule – Gameplay**  **Injuries – Overall**  Determine the effect of the lowering the helmet to initiate contact rule (targeting)) has on lower extremity injuries. | **Injury Definition**  24-h TL  **Measure** Lower extremity injuries.  SRC rate.  Knee injuries resulting in Games lost  Or Injury reserve. | **Lower Extremity**  34.3/1000 AE  **SRC Injuries**  5.5/1000 AE  **Games Lost**  n = 4,011 games  **Injury Reserve**  n = 346 players | **Lower Extremity**  33.6/1,000 AE  **SRC Injuries**  3.3/1,000 AE  **Games Lost**  n = 9,363 games  **Injury Reserve**  n = 745 players | **Lower Extremity RR**  IRR = 0.97 (0.92 to 1.04) p = 0.50  **SRC Injury RR**  IRR = 0.60 (0.50 to 0.73) p = 0.0001  **Games Lost**  p = 0.01  **Injury Reserve RR**  IRR = 1.15 (0.99 to 1.33), p = 0.06 | **Outcomes**  Rule change had intended effect of reducing the rate of SRC.  There was no significant increase to the rate of lower extremity injuries.  **Unintended consequence**  The severity of lower extremity injuries increased as a higher rate of player either lost game time or were placed on IR. | **Limitations**  Public injury reports used to collect the data were only made available two days after a game had been played.  Pre-season games were not assessed as part of this study due to the difference in competition level and inability to track players injuries if they were cut from the team. |
| **American Football**  (United States)  Waller et al. (2024) | Male  Elite  Adult  Teams (n = 32), Games (n = 20/season) | **Retrospective Cohort**  Open access injury data | **Rule – Gameplay**  **Injuries – Head**  Ban on lowering the helmet to initiate contact in the NFL (from 2021 this penalty was group with the unnecessary roughness call). | **Injury Definition**  Diagnosed SRC  **Measure**  SRC rate (per 1,000 plays) | **SRC Rate (2015-17)**  5.37/1,000 competitive plays (95% CI not reported) | **SRC Rate (2018-20)**  4.01/1,000 competitive plays (95% CI not reported)  **SRC Rate (2021-22)**  3.99/1,000 competitive plays (95% CI not reported) | **SRC Rate**  IRR = 0.76 (95% CI not reported)  **SRC Rate (2015-17 vs 2021-22)**  IRR = 0.74 (95% CI not reported) | **Outcomes**  There was a significant difference in injury trends across the years (χ2 = 16, p = <0.01). while year to year changes were not significant.  SRC per play showed a significant decrease from 2015-2022.  When a lowering the helmet penalty was called a player was diagnosed with a concussion 3.5% of the time. | **Limitations**  The sample size was small as the penalty had only been implemented from season 2018.  Due to this penalty now being grouped with the unnecessary roughness call, determining the effect of this specific rule change is impossible.  Due to internal and external factors player may not have reported concussions. |
| **Canadian Football**  (Canada)  Pankow et al. (2025) | Male  Amateur  Adolescent  Teams (n = 2) | **Retrospective cohort**  Video analysis | **Rule – Gameplay**  **Injuries – Head**  Mercy rule (MR), game clock does not stop in the 2nd half when a set score differential has been met (35+ points) | **Injury Definition**  Head impacts  **Measure**  Head impact rate  Head impact rate - 2^nd^ half of games (per team game and per team second half) | **Head impacts**  328.91/team game (313.04 to 345.57)  **2^nd^ Half head impacts**  166.20/team 2^nd^ half (156.75 to 176.22) | **Head impacts**  241.67/team game (199.24 to 293.13)  **2^nd^ Half head impacts**  99.16/team 2^nd^ half (76.81 to 128.03) | **Head impacts**  IRR = 0.73 (0.61 to 0.89)  **2^nd^ Half head impacts**  IRR = 0.60 (0.47 to 0.76) | **Outcomes**  There were significantly less head impacts in MR games with this change being attributed to a reduction in exposure caused by 28% less plays executed.  Player attitudes changing when the MR rule was enforced may have impacted the outcome. Although, when offsetting for the number of player-plays during the MR the influence was minimal. | **Limitations**  The absence of injury data limits the level of evaluation that can be done.  Head impact rates may be underestimated due to obstruction on video. |
| **American Football** (United States)  Westerman et al. (2016) | Male  Amateur  Adult  Teams (n = 68.  Team seasons (n = 153) | **Prospective Cohort**  Injury surveillance forms. | **Rule – Gameplay**  **Injuries - Overall**  Targeting rule -penalising players for direct head-to-head contact. | **Injury Definition**  MA + 24-h TL  **Measure** SRC rate.  Lower extremity. | **SRC Injuries**  2.63/1000 AE  **Lower Extremity**  MA 20.45/1000 AE (19.28 to 21.62)  24-h 10.11/1000 AE (9.39 to 11.04) | **SRC Injuries**  3.52/1000 AE  **Lower Extremity**  MA 23.55/1000 AE (22.23 to 24.87)  24-h 12.38/1000 AE (11.42 to 13.34) | **SRC Injury RR**  IRR = 1.34 (1.08 to 1.66)  **Lower Extremity RR**  MA IRR = 1.15 (1.06 to 1.25)  24-h IRR = 1.21 (1.06 to 1.36) | **Outcomes**  The rate of SRC increased.  SRC increase partially attributed to improved education regarding recognise and remove procedures.  **Unintended consequence**  The targeting rule increased the rate of lower extremity injuries. | **Limitations**  Did not account for player and coaching behaviour changes.  Injury mechanics and event were attributed to the increase in injuries. |
| **American Football** (United States)  Whelan et al. (2023) | Male  Amateur  Adult  KO plays (n = 6,958) | **Retrospective Cohort**  Injury surveillance forms. | **Rule – KO**  **Injuries - Head**  Allow a fair catch to be called up to the 25 yd line. | **Injury Definition**  Clinical Treatment  **Measure**  SRC Rate (Per 1,000 KO plays). | **SRC Injuries** 3.42/1,000 KO plays | **SRC Injuries**  5.31/1,000 KO plays | **SRC Injury RR**  KO IRR = 1.89 (-1.22 to 5.01) | **Outcomes**  The rate of SRC did increase after the rule change, although not significantly.  The rule change did have the intended effect on game play as it increased the quantity of touch backs by 6% (p = <0.001)  KO is a high-risk play and requires further amendments to improve player safety. | **Limitations**  Sample size was limited due to minimal KO occurring within each game.  Results cannot be extrapolated across all levels and competitions. |
| **American Football** (United States)  Baker et al. (2018) | Male  Elite  Adult  Games (n = 256) | **Retrospective Cohort**  Open access injury data. | **Rule – Gameplay**  **Injuries – Lower body** Chop block – penalising players for diving at an opponent’s knees as a method to block. | **Injury Definition**  24-h TL  **Measure** Defensive player Knee Injuries.  Knee injuries resulting in Injury reserve designation. | **Knee Injuries**  2014/15 = 789/1,000 AE  2015/16 = 621/1,000 AE  **Injured Reserve**  2014-16 (n = 131) | **Knee Injuries**  2016/17 = 460/1,000 AE  2017/18 = 621/1,000 AE  **Injured Reserve**  2016-18 (n = 116) | **Knee Injury RR** RR = 0.84 (0.75 to 0.96), p = 0.09  **Injury Reserve RR**  RR = 0.90 (0.72 to 1.13), p = 0.39 | **Outcomes**  Comparing two seasons of pre and two seasons of post rule change showed a reduction in defensive player knee injuries.  No change in injury severity as the quantity of plyers being placed on IR for knee injuries did not decrease. | **Limitations**  Public injury reports used to collect the data lacks detailed information including type, cause, and treatment of injury.  Separate rule changes to the KO procedures and horse collar tackling may have positively influenced the result. |
| **American Football** (United States)  Torg et al. (1990) | Male  Amateur  Mixed  Quantity of player not reported. | **Retrospective Cohort**  Injury surveillance forms.  Open access injury data.  Hospital/medical/insurance records. | **Rule – Tackling**  **Injuries – Upper body**  Removal of headfirst blocking and spear tackling. | **Injury Definition**  72-h TL + hospitalisation  **Measure** Spine Trauma.  Quadriplegia.  Haemorrhage.  Death. | **(/100,000 players)**  **Spine Trauma** HS = 7.72/100,00  College =30.66/100,00  **Quadriplegia**  HS = 2.24/100,000  College = 10.66/100,000  **Haemorrhage** HS = 0.89/100,00  College = 1.33/100,00  **Death**  HS = 0.98/100,00  College = 0.0/100,000 | **(/100,000 players)**  **Spine Trauma** HS = 2.31/100,00  College =10.66/100,00  **Quadriplegia**  HS = 0.73/100,000  College = 0.0/100,000  **Haemorrhage** HS = 1.78/100,00  College = 0.0/100,00  **Death**  HS = 0.10/100,00  College = 0.0/100,000 | **% Change**  **Spine Trauma** HS = -70%  College = -65%  **Quadriplegia**  HS = -82%  College = -100%  **Haemorrhage**  HS = +100%  College = -100%  **Death**  HS = -90%  College = N/A | **Outcomes**  There was a reduction in spinal trauma injuries as a result of the law change.  Improved coaching and tackling techniques may also have contributed to a reduction in injuries. | **Limitations**  Study did not report any limitations. |
| **American Football** (United States)  Ruestow et al. (2015) | Male  Elite  Adult  Special Teams  Teams (n = 32) | **Retrospective Cohort**  Open access injury data. | **Rule – KO**  **Injuries – Overall**  Move KO mark from 30yd to 35yd line. KO Team max 5 yd behind kicker. | **Injury Definition**  24-h TL  **Measure**  Injury rate.  SRC rate  (Per 1,000 kick-off and kick return plays). | **Injuries**  22.0/1,000 KO  26.0/1,000 KR  **SRC Injuries** 4.40/1,000 KO | **Injuries**  10.0/1,000 KO  17.0.4/1,000 KR  **SRC Injuries** 2.20/1,000 KO | **Injury RR**  KO IRR = 0.45 (0.28 to 0.73)  KR IRR = 0.75 (0.41 to 1.08)  **SRC Injury RR**  KO IRR = 0.49 (0.13 to 1.81) | **Outcomes**  Decrease in the rate of injuries and SRC attributed to the KO play.  Rule change had intended in game effect as it increased the quantity of touch backs. | **Limitations**  Injury data from public sources cannot be medically verified.  Data lacked player position information.  Prior injuries were not accounted for. |
| **Rugby Union**  (New Zealand)  Gianotti et al. (2008) | Male  Amateur  Adult  Unavailable | **Retrospective Cohort**  Hospital/medical/insurance records. | **Rule – Scrum**  **Injuries – Upper body** Scrum engagement sequence altered to “crouch, touch, pause, engage”. | **Injury Definition**  7-day TL  **Measure** Spinal Injuries (based on insurance claims) | **Spinal Injuries**  66.0/100,000 forward players | **Spinal Injuries 2007**  52.0/100,000 forward players | **Spinal Injuries RR** IRR = 0.79 (0.53 to 1.18)/100,000 insurance claims | **Outcomes**  The scrum law has likely reduced spinal injury insurance claims however the results were not significant.  Improved education and coaching techniques may have helped to reduce spinal injuries during this time period. | **Limitations**  Only accounted for acute spinal injuries.  Exposure rate of scrums unaccounted for. |
| **Rugby Union** (France)  Reboursiere et al. (2018) | Male and female  Mixed  Mixed  Players – Adults (n = 1,229,468), Adolescents (n = 689,339) over 7 seasons. | **Prospective Cohort**  Hospital/medical/insurance records. | **Rule – Scrum**  **Injuries – Upper body** Scrum engagement sequence altered to “crouch, placing, pause, play”. | **Injury Definition**  Permanent spinal injury (catastrophic)  **Measure** Spinal Injuries (per 100,000 players). | **Spinal Injuries** Adult – 4.5/100,000 players (2.4 to 6.6)  Adolescent – 0.4/100,00 players (0 to 1)  **Scrum Spinal Injuries**  1.1/100,000 players (0.4 to 1.7) | **Spinal Injuries** Adult – 2.2/100,000 players (0.6 to 3.9)  Adolescent – 0.7/100,00 players (0 to 1.4)  **Scrum Spinal Injuries**  0.1/100,000 players (0 to 0.3) | **Spinal Injuries**  Adult IRR = 0.48  Adolescent IRR = 1.75  **Scrum spinal injuries**  IRR = 0.09, p = 0.02 | **Outcomes**  Rule change reduced the overall rate of spinal injuries in the adult game but did not find an effect in the adolescent game.  Catastrophic spinal injuries caused by the scrum reduced from 55% (2006-10) to 9% (2010-13). | **Limitations**  Does not include all catastrophic spinal injuries.  Study could not report incidence to exact playing position due to a lack of data.  Nationwide injury prevention strategies to improve education and coaching also likely to have had a positive effect. |
| **Rugby Union** (England)  Stokes et al. (2021) | Male  Elite  Adult  Matches – Control (n = 90), Intervention (n = 36) | **Prospective Review**  Injury surveillance forms.  Video Analysis. | **Rule – Tackle**  **Injuries - Overall** Reducing the legal tackle height from shoulders to below the armpit. | **Injury Definition**  24-h TL  **Measure** Incidence rate.  SRC Rate. | **Incidence Rate** 71.1/1,000 hours (62.9 to 80.4)  **SRC Rate** 16.9/1,000 hours (13.2 to 21.8)  **Tackler SRC Rate**  6.9/1,000 hours | **Incidence Rate** 70.8/1,000 hours (58.3 to 86.0)  **SRC Rate** 22.2/1,000 hours (15.7 to 31.3)  **Tackler SRC Rate**  13.2/1,000 hours | **Incidence RR**  IRR = 1.00 (0.79 to 1.25), p = 0.97  **SRC RR** IRR = 1.31 (0.85 to 2.01), p = 0.21  **Tackler SRC Rate**  IRR = 1.90 (1.05 to 3.45), p = 0.035 | **Outcomes**  The rule change did not significantly change the rate of injuries or SRC.  Significant increase in SRC for the tackler.  Player behaviour did alter as 15% less tackles were executed above the armpit resulting in fewer head and neck contacts. | **Limitations**  Variation in team selection as the trial games were cup fixtures instead of league matches.  Pitch/ground conditions were unaccounted for.  Stakeholder engagement and preparation time should have been longer prior to the law change trial. |
| **Rugby Union** (South Africa)  Van Tonder et al. (2023) | Male  Amateur  Adult  Matches – Control (n = 101), Intervention (n = 116) | **Cross Sectional**  Injury surveillance forms. | **Rule – Tackle**  **Injuries - Overall** Reducing the legal tackle height from the shoulders to below the armpit. | **Injury Definition** MA + 24-h TL  **Measure** Tackling SRC.  Tackled SRC.  Injury rate  SRC rate | **Tackling SRC**  3.1/1,000 match hours (1.4 to 5.9)  **Tackled SRC**  1.0/1,000 match hours (0.2 to 3.0)  **Incidence Rate**  MA 30.9/1,000 match hours (24.5 to 37.3)  TL 30.6/1,000 match hours (24.2 to 36.9)  **SRC Injuries** TL 8.9/1,000 match hours | **Tackling SRC**  2.6/1,000 match hours (1.2 to 5.0)  **Tackled SRC**  1.5/1,000 match hours (0.5 to 3.4)  **Incidence Rate**  MA 28.4/1,000 match hours (22.8 to 34.1)  TL 24.3/1,000 match hours (19.1 to 29.5)  **SRC Injuries** TL 6.1/1,000 match hours | **Tackle Event SRC RR**  SRC IRR = 0.99 (0.53 to 1.87), p = 0.99  **Incidence RR**  MA IRR = 0.92 (0.7 to 1.2), p = 0.56  TL IRR = 0.79 (0.60 to 1.11), p = 0.13  **SRC Injury RR** TL IRR = 0.69 (0.40 to 1.10), p = 0.20 | **Outcomes**  The tackle event (combination of SRC from being tackled and tackling) showed no reduction in rate.  The rule change did reduce the overall rate of injury and SRC, although the change was not statistically significant.  Injury severity did not differ between the two seasons. | **Limitations**  Limited sample size due to environmental factors such as droughts andCovid-19 pandemic.  Injuries treated out with clinical settings not accounted for. |
| **Rugby Union**  (England)  Roberts et al. (2025) | Male  Amateur  Adolescent  Games – Pre (U15 n = 11, U18 n = 16), Post (U15 n = 10, U18 n = 10), Players (n = 1105), Schools (n = 36), | **Prospective cohort**  Injury surveillance forms.  Video Analysis. | **Rule – Tackle**  **Injuries - Overall** Reducing the legal tackle height from the shoulders to below the armpit. | **Injury Definition**  24-h TL  **Measure**  Injury rate  SRC rate  Tackle SRC | **Injury rate**  U15 24.6/1,000 hours (20.3 to 29.5)  U18 34.6/1,000 hours (31.4 to 38.1)  **SRC rate**  U15 6.4/1,000 hours (4.4 to 9.1)  U18 9.2/1,000 hours (7.6 to 11.1)  **Tackle SRC**  U15 3.7/1,000 hours (2.2 to 5.9)  U18 5.3/1,000 hours (4.1 to 6.8) | **Injury rate**  U15 19.5/1,000 hours (13.5 to 27.3)  U18 38.3/1,000 hours (31.5 to 46.2)  **SRC rate**  U15 9.2/1,000 hours (5.3 to 14.9)  U18 8.7/1,000 hours (5.6 to 12.9)  **Tackle SRC**  U15 5.2/1,000 hours (2.4 to 9.8)  U18 4.9/1,000 hours (2.7 to 8.2) | **Injury RR**  U15 RR = 0.79 (0.53 to 1.17), p = 0.27  U18 RR = 1.11 (0.89 to 1.37), p = 0.34  **SRC RR**  U15 RR = 1.43 (0.73 to 2.70), p = 0.31  U18 RR = 0.95 (0.59 to 1.47), p = 0.82  **Tackle SRC RR**  U15 RR = 1.39 (0.55 to 3.25)  U18 RR = 0.92 (0.48 to 1.65) | **Outcomes**  There was no significant change in the rate of injuries, SRC or SRC caused by the tackle event.  The video analysis showed the law change was successful in changing tackle technique behaviour as intended.  The low penalisation rate at U18 level may be linked to the lack of SRC reduction as the deterrent was not enforced enough. | **Limitations**  There was a longer period of data collection pre-law versus law change.  The study did not examine any contextual information from stakeholders relating to knowledge and attitudes of the law change.  The participating school were mostly high performing, and further work should be done to assess players of varying ability. |
| **Rugby Union**  (Scotland)  Gornall et al. (2025) | Male  Amateur  Adult  Games – Pre (n = 30), Post (n = 30) | **Retrospective cohort**  Video Analysis | **Rule – Tackle**  **Head contacts** Reducing the legal tackle height from the shoulders to the base of the sternum. | **Injury Definition**  Tackler (T) and ball-carrier (BC) Head-to-head and head-to-shoulder contact.  **Measure**  Incidence rate | **Head-to-head**  T = 0.44/100 tackle actions (0.31 to 0.57)  BC = 0.38/100 tackle actions (0.25 to 0.50)  **Head-to-shoulder**  T = 1.93/100 tackle actions (1.65 to 2.21)  BC = 2.65/100 tackle actions (2.32 to 2.97) | **Head-to-head**  T = 0.24/100 tackle actions (0.14 to 0.34)  BC = 0.23/100 tackle actions (0.13 to 0.33)  **Head-to-shoulder**  T = 1.36/100 tackle actions (1.20 to 1.60)  BC = 1.81/100 tackle actions (1.53 to 2.08) | **Head-to-head RR**  Tackler RR = 0.55 (0.33 to 0.92)  Ball-carrier RR = 0.61 (0.36 to 1.05)  **Head-to-shoulder RR**  Tackler RR = 0.71 (0.56 to 0.89)  Ball-carrier RR = 0.68 (0.56 to 0.83) | **Outcomes**  There was a significant decrease in head-to-head and head-to-shoulder contacts for the tackling player.  There was a significant decrease in head-to-shoulder contacts for the ball-carrier.  Tacklers were bent at the waist and contacted the ball-carriers lower body more often during the law change seasons. | **Limitations**  The study only evaluated the top amateur division in the country and results may be different in lower leagues.  No injury surveillance data was captured therefore establishing the rate of concussion was not possible. |
| **Rugby Union** (Scotland)  Walton et al. (2025) | Female  Amateur  Adult  Games – Pre (n = 16), Post (n = 18), Players (n = 208), Teams (n = 10) | **Prospective Review**  Injury surveillance forms.  Video Analysis. | **Rule – Tackle**  **Injuries – overall and Head contacts** Reducing the legal tackle height from the shoulders to the base of the sternum. | **Injury Definition**  24-h TL. Head-to-head and head-to-shoulder contact.  **Measure**  Tackler SRC  Tackled SRC  SRC rate  Injury rate | **Tackler SRC**  2.83/1,000 hours (1.04 to 6.17)  **Tackled SRC**  1.89/1,000 hours (0.51 to 4.84)  **SRC Rate**  7.08/1,000 hours (3.97 to 11.68)  **Injury rate**  25.98/1,000 hours (19.57 to 33.81) | **Tackler SRC**  0.52/1,000 hours (0.01 to 2.91)  **Tackled SRC**  4.70/1,000 hours (2.15 to 8.92)  **SRC Rate**  7.83/1,000 hours (4.38 to 12.92)  **Injury rate**  17.76/1,000 hours (12.30 to 24.81) | **Tackler SRC RR**  RR = 0.18 (0.004 to 1.52)  **Tackled SRC RR**  RR = 2.49 (0.69 to 11.06)  **SRC RR**  RR = 1.11 (0.50 to 2.43)  **Injury RR**  RR = 0.68 (0.43 to 1.07) | **Outcomes**  There was no significant change in the rate of concussion related to the tackle event.  The overall rate of injuries decreased but not significantly.  Tacklers behaviour did align with the intention of the law change whereby less contact was made to the ball-carrier head/neck and upper torso. And less head-to-head proximity was identified. | **Limitations**  The evaluation only accounted for a single trial season and further analysis may be required to determine the impact of this law change.  The sample size was small and therefore results must be interpreted with caution.  A total of 34 games were analysed and therefore no randomised selection of games was able to be made. |
| **Football**  (Germany)  Beaudouin et al. (2019) | Male  Elite  Adult  Games (n = 34/team, + national/international competitions) | **Retrospective Cohort**  Open access injury data.  Video analysis. | **Rule – Heading**  **Injuries - Head**  Penalising intentional elbow-head contact with a red card. | **Injury Definition**  MA + 24-h TL  **Measure** Head Injuries.  SRC rate. | **Head Injuries**  2.63/1,000 hours (2.29 to 3.03)  **SRC Injuries** 0.67/1,000 hours (0.51 to 0.88) | **Head Injuries** 1.87/1,000 hours (1.58 to 2.21)  **SRC Injuries** 0.48/1,000 hours (0.34 to 0.66) | **Head Injury RR**  IRR = 0.71 (0.57 to 0.86), p = 0.002  **SRC Injury RR**  IRR = 0.71 (0.46 to 1.09), p = 0.12 | **Outcomes**  Head to elbow injuries reduced by 29% as a result of the rule change.  There was a significant reduction in head injuries and substantial decline in SRC injuries.  **Unintended consequence**  Free balls caused more injury compared to in possession heading. | **Limitations**  Interpretation of head injuries may differ, resulting in unrecorded incidents.  Additional rule changes may have impact the IR. |
| **Football** (Germany)  Beaudouin et al. (2020) | Male  Elite  Adults  Teams (n = 18), players (n = 570) | **Retrospective Cohort**  Open access injury data  Video analysis | **Rule – Heading injuries - Head**  Penalising intentional elbow-head contacts. | **Injury Definition**  MA + 24h TL  **Measure**  Head injuries  Elbow-to-head | **Head Injuries**  2.63/1,000 player hours  **Elbow-to-head**  n = 35 | **Head Injuries**  1.87/1,000 player hours  **Elbow-to-head**  n = 27 | **Head Injuries RR**  RR = 0.71 (95% CI not reported)  **Elbow-to-head**  23% less | **Outcomes**  The rule change has potentially led to a reduction in elbow-to-head injuries.  Only 31% of head-to-elbow injuries were penalised by the referee with no increase in red cards.  Free ball situations produce the highest risk of head injury at 81%. | **Limitations**  The RR and IRR were not calculated for the observational analysis due to a large number of missing video sequences.  The retrospective nature of the study meant assessing variables such as tactics and habits was not possible. |
| **Football**  (Norway)  Bjørneboe et al. (2013) | Male  Elite  Adult  Contact Incidents (n = 1421) | **Prospective Review**  Injury surveillance forms.  Video Analysis. | **Rule – Heading**  **Injuries – Head** Red card enforcement for elbow to head contact and late tackling. | **Injury Definition**  MA  **Measure** Contact Incidents.  Head contact. | **Head Contact**  28.5/1000 hours (24.8 to 32.3)  **Contact Incidents**  109.6/1000 hours (102.3 to 116.9) | **Head Contact**  23.2/1,000 hours (19.9 to 26.6)  **Contact Incidents**  107.7/1,000 hours (100.5 to 114.9) | **Head Contact RR** IRR = 0.81 (0.67 to 0.99)  **Contact Incidents RR**  IRR = 0.98 (0.89 to 1.08) | **Outcomes**  Head to elbow incidents decreased as a result of the new rule.  Overall contact incidents caused by dangerous and late tackling did not decrease. | **Limitations**  Not all injuries caused by a contact event were recorded by medical personnel.  35 injuries were not captured on video and therefore unassessed. |
| **Football**  (Japan)  Shibukawa et al. (2024) | Male  Elite  Adults  Teams (n = 18), matches (n = 306) | **Retrospective Cohort**  Video analysis | **Rule – Aerial collisions – Head**  Allow outfield players into the box when restarting from a goal kick (lowering free ball aerial collisions from long goal kicks) | **Injury Definition**  **Aerial collisions**  **Measure**  Aerial collision (AC)  Goal-kick AC  Goal-kick pass AC | **Aerial Collisions**  Mean = 37.14/match ($\pm$ 12.07)  **Goal Kick AC**  Mean = 6.08/match ($\pm$3.14)  **Goal-kick pass AC**  Mean = 1.59/match ($\pm$1.50) | **Aerial Collisions**  Mean = 28.62/match ($\pm$ 9.07)  **Goal Kick AC**  Mean = 3.92/match ($\pm$2.47)  **Goal-kick pass AC**  Mean = 0.77/match ($\pm$0.97) | **Aerial Collisions**  23% less AC, p = <0.01  **Goal Kick AC**  36% less GLK-AC, p = <0.01  **Goal-kick pass AC**  52% less GKP-AC, p = <0.01 | **Outcomes**  The decrease in long goal kicks and therefore less AC may lead to a reduction in the rate of head injuries and SRC.  The frequency of AC related fouls decreased after implementing the rule change.  The rule change may have led to tactical changes that helped decrease the overall rate of AC. | **Limitations**  It was not possible to assess the rate of AC that led to injury in this analysis.  Assessing the impact of this rule in other contexts and competition levels is important to determine if similar results are found. |
| **Football**  (United States)  Kriz et al. (2022) | Male and female  Amateur  Adolescent  Contact Injuries (n = 901) | **Retrospective Cohort**  Open access injury data.  NGB injury database. | **Rule – Sanctions**  **Injuries - Overall** Yellow card policy - Suspension for accumulation of yellow card fouls. | **Injury Definition**  MA + 24h TL  **Measure** Contact Injuries.  SRC rate. | **Contact Injuries**  Male = 20.7/10,000 AE  Female = 28.1/10,000 AE  **SRC Injuries** M+F = 26.8/10,000 AE | **Contact Injuries**  Male = 22.8/10,000 AE  Female = 28.7/10,000 AE  **SRC Injuries** M+F = 27.4/10,000 AE | **Contact Injury RR**  (M) IRR = 1.10 (0.99 to 1.22)  (F) IRR = 1.02 (0.92 to 1.13)  **SRC Injury RR** M + F IRR = 1.03 (0.88 to 1.22) | **Outcomes**  IR did not change during the 13-year YCP period for male or female athletes.  The rule is highly dependent on officials enforcing the rule on a regular and consistent basis. | **Limitations**  Data was only available from schools with athletic trainers present, therefore reducing the sample size.  IR may be underreported as reporting was only done if MA from an athletic trainer was sought >1 day after the injury incident. |
| **Football**  (United States)  Lalji et al. (2020) | Male and female  Amateur  Adolescent  Injuries - Control (n = 3670), Intervention (n = 3826) | **Retrospective Cohort**  Hospital/medical/insurance records. | **Rule – Heading**  **Injuries - Head**  Disallowing heading of the ball for players between 10-13 years. | **Injury Definition**  Clinical Treatment  **Measure**  SRC Injuries (quantity of SRC injuries, and % they made-up of all injuries).  Injuries. | **SCR Injuries**  Male (n = 150, 7%)  Female (n = 129, 8.5%)  **Injuries**  Male (n = 2,000)  Female (n = 1,391) | **SCR Injuries** Male (n = 200, 8.24%)  Female (n = 152, 10.86%)  **Injuries**  Male (n = 2,226)  Female (n = 1,248) | **SCR Injuries**  Pre vs Post OR = 1.286 (1.09 to 1.52), p = 0.003  **Likelihood of SRC injury M v F**  OR = 1.242 (1.05 to 1.47), p = 0.011 | **Outcomes**  SRC in relation to all other injuries increased significantly after the rule change was implemented.  Female athletes were more likely to have a SRC in relation to all other injuries compared to males.  SRC increase may be attributed to improved education regarding recognise and remove. | **Limitations**  The study sample size was limited (100 hospitals included in the data).  Injuries treated out with clinical settings were not accounted for in the study.  Potential for SRC to be underreported in this setting if athletes do not seek professional care for SRC. |
| **Football**  (Spain)  Qureshi et al. (2025) | Male  Elite  Adult  Injuries – Pre (n = 1,268), Post (n = 711) | **Retrospective Cohort**  Open access injury data | **Rule – Gameplay**  **Injuries – Overall**  Increase the maximum number of substitutions in a competitive game from 3 to 5. | **Injury Definition**  MA + 24h TL  **Measure**  Injury rates  Muscle injuries  Multiple injuries (re-injury) | **Injuries**  0.94/player seasons (95% CI not reported)  **Muscle Injuries**  N = 797  **Multiple Injuries**  N = 595 | **Injuries**  0.57/player seasons (95% CI not reported)  **Muscle Injuries**  N = 399  **Multiple Injuries**  N = 278 | **Injuries**  Number of individual injured players 16% less, p = <0.01  **Muscle Injuries**  OR = 0.72 (95% CI 0.59 to 0.87), p = <0.01  **Multiple Injuries**  OR = 0.68 (95% CI 0.56 to 0.82), p = <0.01 | **Outcomes**  The number of individual injured players decreased significantly when the rule was introduced.  Both the odds of muscle injuries and a player having multiple injuries in a single season decreased.  Further work should be done in different context to validate and corroborate these findings as this evaluation was only conducted in elite top division Spanish football. | **Limitations**  The retrospective design with a reliance on open-source injury data does hold reliability limitations.  Study design limited the opportunity to look at casual relationships and injury trends.  The study could not account for confounding factors including training protocols, injury prevention strategies, injury reporting and player roster changes. |
| **Australian Rules Football**  (Australia)  Orchardet al. (2009) | Male  Elite  Adult  Players (n = 4,092) | **Retrospective Cohort**  Injury surveillance forms. | **Rule – KO**  **Injuries – Lower body** Limit the run-up of ruck men at the centre bounce (KO). | **Injury Definition**  Game Loss  **Measure** Posterior cruciate ligament Injuries (Knee). | **PCL Injuries**  Centre Bounce PCL 3.7/10,000 player hours  All PCL 12.9/10,000 player hours | **PCL Injuries**  Centre Bounce PCL 0.6/10,000 player hours  All PCL 5.9/10,000 player hours | **PCL Injury RR**  Centre Bounce RR = 0.16 (0.04 to 0.69), p = <0.01  All PCL RR = 0.45 (0.28 to 0.75), p = <0.01 | **Outcomes**  The rule change significantly reduced PCL injuries at KO as well as across the wider game.  Longitudinal injury surveillance was key to identifying the injury mechanism and assessing the impact of the rule change. | **Limitations**  The study was unable to attribute which exact factors resulted in reducing the rate of PCL injuries (e.g. reduced trauma from rucks, decrease in ground hardness). |
| **Australian Rules Football**  (Australia)  Orchard et al. (2014) | Male  Elite  Adult  Unavailable | **Retrospective Cohort**  Injury surveillance forms. | **Rule – Multiple**  **Injuries - Overall**  8 rule changes introduced between 2004 to 2013:  1. Knee PCLs (05)  2. Head/neck (07)  3. Lower limb (11)  4. SRC (11)  5. Head/neck (11)  6. Knee PCLs (13)  7. SRC (13)  8. Leg fractures + Ankle injuries (13) | **Injury Definition**  Game Loss  **Measure** Knee PCL Injuries.  Head/Neck.  Groin/Hamstring. SRC.  Leg fractures. | **IR not reported in text** | **IR not reported in text** | **Incidence RR**  **Knee PCLs (2005)** IRR = 0.16 (0.04 to 0.69), p = <0.01  **Head/Neck (2007)**  IRR = 0.72 (0.57 to 0.91)  **Groin & Hamstring (2011)**  IRR = 0.76 (0.62 to 0.93)  IRR = 0.81 (0.70 to 0.93)  **SRC (2011)**  IRR = 2.25 (1.53 to 3.31)  **Head/Neck (2011)**  IRR = 1.78 (1.39 to 2.27)  **Knee PCLs (2013)**  IRR = 1.04 (0.56 to 1.93)  **SRC (2013)**  IRR = 1.17 (0.77 to 1.80)  **Leg Fractures (2013)**  IRR = 1.06 (0.63 to 1.78) | **Outcomes**  The rule changes had varied impacts on reducing the rate of injury.  limiting the run up of ruck men to reduce PCL injuries was highly effective.  The introduction of more conservative SRC guidelines may have influenced the rate of SRC.  **Unintended consequence**  Penalising head contact may incentivise players to place their head in dangerous areas to gain a free kick advantage. | **Limitations**  Game loss injury definition may exclude a large quantity of injuries including SRC.  Rule changes may not have been implemented for long enough to evaluate them in a holistic manner.  The study does not account for umpiring changes regarding the interpretation and enforcement of existing rules. |
| **Rugby League**  (Australia)  Gabbett et al. (2005) | Male  Elite  Adult  Players –  Control (n = 87), Intervention (n = 52) | **Prospective Cohort**  Injury surveillance forms. | **Rule – Gameplay**  **Injuries – Overall**  Teams limited to 12 interchange replacement players rather than unlimited. | **Injury Definition**  7-day TL  **Measure** Incidence rate. | **Incidence Rate** 72.5/1,000 hours (58.2 to 86.8) | **Incidence Rate** 51/1,000 hours (33.8 to 68.1) | **Incidence RR** IRR = 0.70 (0.65 to 0.75) p = <0.05 | **Outcomes**  Increasing players fatigue levels reduced the intensity of gameplay, resulting in a decreased rate of muscular strains and running injuries. | **Limitations**  3 season study offers a limited sample size.  Additional work is required to determine the long-term effects of this rule change. |
| **Lacrosse**  (United States)  Guillaume et al. (2021) | Male  Amateur  Adolescent  Injuries (n = 2,208) | **Retrospective Cohort**  Injury surveillance forms. | **Rule – BC**  **Injuries - Overall** Penalise intentional hits to the head and bodychecking defenceless players. | **Injury Definition**  24-h TL  **Measure** Injuries.  BC injuries.  SRC Rate. | **Incidence Rate** Injuries Competition  4.45/1,000 AE (4.08 to 4.81)  BC Competition  1.35 /1,000 AE (1.14 to 1.55)  Injuries Practice  1.29 /1,000 AE (1.16 to 1.42)  BC Practice  0.21/1,000 AE (0.16 to 0.26)  **SRC Rate** 0.18 /1,000 AE | **Incidence Rate** Injuries Competition  4.21/1,000 AE (3.89 to 4.54)  BC Competition  1.23 /1,000 AE (1.06 to 1.41)  Injuries Practice  1.09 /1,000 AE (0.98 to 1.20)  BC Practice  0.15 /1,000 AE (0.11 to 0.20)  **SRC Rate**  0.18 /1,000 AE | **Incidence RR**  Injuries Competition  IRR = 0.95 (0.85 to 1.06)  BC Competition  IRR = 0.92 (0.75 to 1.13)  Injuries Practice  IRR = 0.85 (0.74 to 0.98)  BC Practice  IRR = 0.73 (0.51 to 1.05)  **SRC Rate RR** IRR = 0.98 (0.60 to 1.6) | **Outcomes**  The rule changes showed a reduction in checking injuries in practice environments.  Injuries in game settings were not reduced significantly by the rule change.  The rule change did not reduce SRC injuries significantly.  SRC remaining unchanged was partially attributed to improved education regarding recognise and remove procedures. | **Limitations**  Further investigation is required to determine which rule had a greater effect on the IR.  Data only available from schools with athletic trainers therefore limiting the sample size of the study. |
